# Deletion of CD36 exhibits limited impact on normal hematopoiesis and the leukemia microenvironment

**DOI:** 10.1186/s11658-023-00455-8

**Published:** 2023-05-24

**Authors:** Yiting Meng, Mateusz Pospiech, Atham Ali, Ritu Chandwani, Mary Vergel, Sandra Onyemaechi, George Yaghmour, Rong Lu, Houda Alachkar

**Affiliations:** 1 Department of Clinical Pharmacy, School of Pharmacy, University of Southern California, Los Angeles, CA 90089 USA; 2 Department of Pharmacology and Pharmaceutical Science, School of Pharmacy, University of Southern California, Los Angeles, CA 90089 USA; 3 Keck School of Medicine, University of Southern California, Los Angeles, CA 90033 USA; 4 Division of Hematology, Department of Medicine, University of Southern California Norris Comprehensive Cancer Center, Los Angeles, CA 90033 USA; 5 USC Norris Comprehensive Cancer Center, University of Southern California, Los Angeles, CA 90033 USA

**Keywords:** CD36, Knockout, Hematopoiesis, T Cells, Acute myeloid leukemia, Mouse

## Abstract

**Background:**

CD36 has been identified as a potential therapeutic target both in leukemic cells and in the tumor immune microenvironment. In acute myeloid leukemia (AML), we found that APOC2 acts with CD36 to promote leukemia growth by activating the LYN-ERK signaling. CD36 also plays a role in lipid metabolism of cancer associated T-cells leading to impaired cytotoxic CD8^+^ T-cell and enhanced T_reg_ cell function. To establish CD36 as a viable therapeutic target in AML, we investigated whether targeting CD36 has any detrimental impact on normal hematopoietic cells.

**Methods:**

Differential expression data of *CD36* during human and mouse normal hematopoiesis were examined and compared. Cd36 knockout (Cd36-KO) mice were evaluated for blood analysis, hematopoietic stem cells and progenitors (HSPCs) function and phenotype analyses, and T cells in vitro expansion and phenotypes in comparison with wild type (WT) mice. In addition, MLL-PTD/FLT3-ITD leukemic cells were engrafted into Cd36-KO and WT mice, and leukemia burden was compared between groups.

**Results:**

RNA-Seq data showed that *Cd36* expression was low in HSPCs and increased as cells matured. Phenotypic analysis revealed limited changes in blood count except for a slight yet significantly lower red blood cell count and hemoglobin and hematocrit levels in Cd36-KO mice compared with WT mice (P < 0.05). In vitro cell proliferation assays of splenocytes and HSPCs from Cd36-KO mice showed a similar pattern of expansion to that of cells from WT mice. Characterization of HSPCs showed similar percentages of the different progenitor cell populations between Cd36-KO with WT mice. However, Cd36-KO mice exhibited ~ 40% reduction of the number of colonies developed from HSPCs cells compared with WT mice (P < 0.001). Cd36-KO and WT mice presented comparably healthy BM transplant in non-competitive models and developed similar leukemia burden.

**Conclusions:**

Although the loss of Cd36 affects the hematopoietic stem cell and erythropoiesis, limited detrimental overall impact was observed on normal Hematopoietic and leukemic microenvironments. Altogether, considering the limited impact on normal hematopoiesis, therapeutic approaches to target CD36 in cancer are unlikely to result in toxicity to normal blood cells.

**Supplementary Information:**

The online version contains supplementary material available at 10.1186/s11658-023-00455-8.

## Background

CD36 was initially identified as an 88-kDa platelet glycoprotein IV meditating thrombospondin-1 binding in platelets [1, 2], and later was discovered as a fatty acid translocase and a scavenger receptor participating in lipid metabolism [3, 4]. CD36 has been largely studied in angiogenesis [5], atherosclerosis [6], metabolic disorders [7], and inflammation [8] due to its role in the mediation of fatty acid uptake. CD36 has gained an interest in recent years as a therapeutic target in cancer immunotherapy because of its role in T cell lipid metabolism leading to impaired CD8^+^ T cell [9] and enhanced T_reg_ cell function [10] within the tumor or leukemia microenvironment. CD36 has also been shown to play a role in acute myeloid leukemia (AML). We have recently reported that upregulated APOC2 cooperates with CD36 contributing to promote leukemia growth by activating the LYN-ERK signaling mediated metabolic activities of leukemic cells [11]. Consistently, the knockdown of CD36 or treatment with anti-CD36 antibody reduced the leukemia progression and promoted the overall survival of AML xenograft mice [11]. Altogether, targeting CD36 directly on the leukemic cells or within the leukemia immune microenvironment present a potential therapeutic strategy to treat AML and potentially other malignancies. However, CD36 is also present on various types of normal cells, including monocytes, macrophages, endothelial cells, adipocytes, and platelets and contribute to their normal functions [12]. Further investigation is needed to determine if targeting CD36 could have any negative effects on normal hematopoiesis, in order to fully develop the therapeutic strategy of targeting CD36 in AML.

## Materials and methods

### *CD36* gene expression analysis of public RNA-Seq datasets

Data of the *CD36* mRNA expression levels in both human and mouse hematopoietic cells were obtained from the Haemosphere public database (https://www.haemosphere.org/) [13] and the BloodSpot public database (https://servers.binf.ku.dk/bloodspot/) [14]. We downloaded the GSE60101 dataset [15] from BloodSpot on April 18th, 2022, to analyze the mouse normal RNA-seq data and generate the scatter plot, which presented the *Cd36* expression level in the normal mouse hematopoietic system. We next curated *CD36* mRNA expression in human normal hematopoietic cells from dataset GSE115736 [13] downloaded on June 8th, 2022, from Haemosphere public database. The *CD36* expression values from the human normal RNA-Seq dataset were presented as log2 transformed transcripts per million (TPM). We also analyzed GSE116177 [13] RNA-Seq data of *Cd36* gene expression level in mouse hematopoiesis, downloaded on June 10th, 2022, from Haemosphere to compare the difference of *Cd36* expression patterns in mouse and human. To further validate the gene expression patterns, additional datasets were obtained. The *Cd36* expression in mouse normal hematopoietic cells from datasets GSE14833 [16] and GSE6506 [17, 18] and *CD36* mRNA levels in normal human hematopoiesis from datasets GSE17054 [19], GSE19599 [20], GSE11864 [21], and E-MEXP-1242 [22] were downloaded on Feb 26th, 2023, from BloodSpot.

### Cd36 knockout and wild type mice

All animal studies were approved by the Institution for Animal Care and Use Committee (IACUC) of USC. Both males and females mice were included in the studies. To investigate the role of Cd36 in normal mouse hematopoiesis, we studied B6.129S1-*Cd36*^*tm1Mfe*^/J Cd36 knockout (RRID:IMSR_JAX:019006) (Cd36-KO) and C57BL/6 J control (RRID:IMSR_JAX:000664) (WT) mouse model obtained from Jackson laboratory. The Cd36-KO mice carry a neomycin cassette in opposite transcription orientation disrupting exon 3 to silence *Cd36* expression [23]. All Cd36-KO and WT mice were placed on a regular laboratory diet (PicoLab Cat# 5053).

### Genotyping

DNA was extracted from Cd36-KO and C57BL/6 J mice liver cells using DNeasy Blood and Tissue Kit (Qiagen Cat# 69504). NanoDrop One was used to quantify DNA concentration and quality. Equal amounts of DNA were then amplified using WT and Cd36-KO reverse primers respectively combined with a common forward primer. The genotyping primers were designed according to Jackson’s Laboratory protocol and ordered from IDT. Taq 2 × master mix (NEB Cat# M0270L) PCR protocol was followed with following cycling conditions: initial denaturation at 95 °C for 30 s, 34 cycles of 95 °C for 30 s, 57.7 °C for 60 s and 68 °C for 40 s; final extension at 68 °C for 5 min. 1% agarose gel in the TAE buffer was then prepared using ethidium bromide. The PCR amplified products were next analyzed by gel electrophoresis, and the bands were visualized using iBright FL1000 Gel imager (ThermoFisher).

### Quantitative polymerase chain reaction (qPCR) analysis

The bone marrow, spleen, and liver tissues were collected from each of Cd36-KO and WT mice (n = 6 mice per group). RNA extraction was performed using TRIzol™ reagent following manufacturer’s protocol (Invitrogen Cat# 15596018). Equal amounts of RNA were used for cDNA synthesis using Superscript IV reverse transcriptase following the manufacturer's protocol (Invitrogen Cat# 18091050). The real-time quantitative PCR (qPCR) was performed using Taqman assay to quantify the *Cd36* gene (Taqman Cat# Mm00432403_m1) and reference housekeeping gene *B2m* (Taqman Cat# Mm00437762_m1) in mouse. The qPCR assay with 10 μL reaction solutions was performed using the ABI QuantStudio 12 K Flex Real-Time PCR system (Applied Biosystems). To check reproducibility, each assay was performed with technical triplicates. The relative transcripts levels of *Cd36* were normalized to *B2m* and calculated using the 2^−∆∆CT^ method.

### Flow cytometry analysis and antibodies

To assess changes in the main T cell phenotypes between Cd36-KO and WT mice, mice spleen tissues were collected for flow cytometry analysis. Splenocytes were stained with CD3 antibody with Percp-Cyanine 5.5-fluorophore (ThermoFisher Cat# 53-0032-82), CD4 antibody with PE-Cyanine 7-fluorophore (ThermoFisher Cat# 25-0042-82), CD8 antibody with PE-eFluor 610-fluorophore (ThermoFisher Cat# 61-0081-82), and CD25 antibody with Super Bright 436-fluorophore (ThermoFisher Cat# 62-0251-82). Cell surface Cd36 expression levels in mice tissues including BM, spleen, blood, and liver were also assessed by flow cytometry using anti-Cd36 antibody with PE-fluorophore (BD System Cat# 562702). In addition, CD45.1 with APC-fluorophore (Invitrogen Cat# 17-0453-81) and CD45.2 with FITC-fluorophore (Invitrogen Cat# 11-0454-81) flow antibodies were used to stain mice cells from different tissues for monitoring BM transplant and leukemia engraftment progression. For HSPCs phenotyping, we used an antibody cocktail composed of the following: PE anti-mouse CD150 (SLAM) antibody (Biolegend Cat# 115904), PE/Cyanine5 anti-mouse CD135 antibody (Biolegend Cat# 135312), CD45.1 monoclonal antibody (A20), PerCP-Cyanine5.5 (Invitrogen, Cat# 45-0453-82), Ly-6A/E (Sca-1) monoclonal antibody (D7), PE-Cyanine7 (Invitrogen, Cat# 25-5981-82), CD34 monoclonal antibody (RAM34), eFluor 660 (Invitrogen, Cat# 50-0341-82), CD117 (c-Kit) monoclonal antibody (2B8), APC-eFluor 780 (Invitrogen, Cat# 47-1171-82), brilliant violet 510 anti-mouse CD127 (IL-7Rα) antibody (Biolegend Cat# 135033), CD11b monoclonal antibody (M1/70), eFluor 450 (Invitrogen Cat# 48-0112-82), Ly-6G/Ly-6C monoclonal antibody (RB6-8C5), eFluor 450 (Invitrogen Cat# 48-5931-82), CD19 monoclonal antibody (eBio1D3 (1D3)), eFluor 450 (Invitrogen Cat# 48-0193-82), CD45R (B220) monoclonal antibody (RA3-6B2), eFluor 450 (Invitrogen Cat# 48-0452-82), CD8a monoclonal antibody (53-6.7), eFluor 450 (Invitrogen Cat# 48-0081-82), CD4 monoclonal antibody (GK1.5), eFluor 450 (Invitrogen Cat# 48-0041-82), CD3 monoclonal antibody (17A2), eFluor 450 (Invitrogen Cat# 48-0032-82), TER-119 monoclonal antibody (TER-119), eFluor 450 (Invitrogen Cat# 48-5921-82), brilliant violet 605 streptavidin (Biolegend Cat# 405229), CD48 monoclonal antibody (HM48-1), and FITC (Invitrogen Cat# 11-0481-82).

### Proliferation assay

In vitro viability assays of T cells (splenocytes) and hematopoietic stem cells and progenitors (bone marrow) collected from one Cd36-KO and one WT mice were performed over ten days of cell culture. We started with ~ 1.2 × 10^7^cells for 3 replicates per group and labeled the cells with 2 $\mathrm{\mu M}$ CellTrace™ CFSE cell tracking dye with alexa fluor 488 fluorophore (Invitrogen Cat# C34554). The CFSE labeled mice primary bone marrow cells were cultured in 20% FBS-RPMI (ThermoFisher Cat# 11875093) supplemented with stem cell factor (SCF) (Peprotech Cat# AF-250-03) and Thrombopoietin (TPO) (Mitenyl Biotec Cat# 130-094-083) cytokines and primary splenocytes were cultured in the same cell growth medium supplemented with phytohemagglutinin (PHA) (ThermoFisher Cat# 00-4977-93) and interleukin-2 (IL-2) (RD System Cat# 402-ML). Trypan blue cell counting assay (ThermoFisher Cat# 15250061) with three replicates was also performed each day to obtain the total number of viable cells per mL of cell suspension medium. Cell proliferation was also monitored by tracking CellTrace™ CFSE dye dilution measured by flow cytometry every other day.

### Colony forming cell (CFC) assay

Mouse bone marrow cells were plated at about 50,000 cells in 1 mL mouse methylcellulose complete media (BD system Cat# HSC007) in a suspension culture plate with 35 mm diameter. The colonies were counted on the 8th day of in vitro cell culture. To confirm reproducibility, CFC assay was repeated with BM cells from three different donor mice three times with two replicates for the 1st and 2nd experiment separately and four replicates for the 3rd experiment for each of the samples in the study of hematopoietic stem cells.

### Hematological laboratory analysis

We collected about 300 μL mouse heart blood in 2% EDTA (Invitrogen Cat# 15575-038) from Cd36-KO and WT mice (n = 6 mice per group). The collected mouse blood samples were sent to Abid Diagnostic for complete blood count (CBC) analysis. The hematological analysis includes white blood cell (WBC) count, red blood cell (RBC) count, hemoglobin (Hb), hematocrit (HCT)/packed cell volume (PCV), mean corpuscular volume (MCV), mean corpuscular hemoglobin (MCH), mean corpuscular hemoglobin concentration (MCHC) and platelet (Autom). Also, the absolute value and percentage of neutrophil, lymphocytes, monocytes, eosinophils, and basophils were evaluated.

### HSPCs population analysis

Both fresh (from n = 4 female mice per group) and frozen (from n = 2 male mice per group) BM cells collected from the femur of WT and Cd36-KO mice were used for HSPCs population assay. The HSPCs from mouse BM were selected and enriched by CD117 MicroBeads (MiltenyBiotech Cat#130-091-224) and Anti-Rat IgG MicroBeads (MiltenyBiotech Cat#130-048-501) with magnet (MiltenyBiotech, LS Column Cat#130-042-401). Both enriched and unenriched HSPCs were stained by a cocktail of mixed antibodies and then resuspended in propidium iodide (PI) medium for running flow cytometry analysis by Aria II (BD Biosciences, San Jose, CA). We used flow cytometry to identify the percentages of enriched lineage-negative (Lin^−^) BM cells, KLS (Lin^−^ IL7R^−^ Ckit^+^ Sca^+^), common lymphoid progenitors (CLP) (Lin^−^ IL7R^+^), the myeloid progenitor populations (Lin^−^ IL7R^−^ Ckit^+^ Sca^−^), multipotent common myeloid progenitor (CMP) (FcgR^−^ CD34^+^), and granulocyte-monocyte progenitor (GMP) (FcgR^+^), and megakaryocyte-erythrocyte progenitor (MEP) (FcgR^−^ CD34^−^) cells.

### BM homing and engraftment studies

To examine the impact of *Cd36* deletion on the ability of HSPCs to home to the bone marrow, 2.0 × 10^7^ to 2.4 × 10^7^ BM cells were collected from the femur of CD45.2^+^ either WT mice or Cd36-KO mice and transplanted via tail vein injection into four CD45.1^+^ recipient 5-month-old female B6.SJL-*Ptprc*^*a*^* Pepc*^*b*^/BoyJ mice (RRID:IMSR_JAX:002014) irradiated with two doses of 450 cGy within 24 h, separately. Approximately 15 h after transplant, all recipient mice were sacrificed, the BM cells were collected and CD45.2^+^ cells in the BM were analyzed by flow cytometry. The homing assay experiment was also repeated by using CD45.1^+^ recipient 3 to 5-month-old male mice with two mice injected with CD45.2^+^ WT BM cells versus another four mice injected with CD45.2^+^ Cd36-KO BM cells.

To assess the impact of *Cd36* deletion on BM engraftment, 2.4 × 10^7^ BM cells collected from CD45.2^+^ either WT or CD36-KO mice were injected intravenously into five 6- to 8-week-old female CD45.1^+^ mice irradiated by single dose of 450 cGy, separately. Five weeks post-transplant, mice were sacrificed, and the bone marrow, spleen, and blood were collected for flow cytometry analysis to assess the percentage of CD45.2^+^ cells.

We also performed a competitive repopulation assay. Cells collected from the bone marrow of CD45.1^+^ WT were mixed with cells collected from the bone marrow of CD45.2^+^ Cd36-KO mice at 1:1 ratio to make the mixture of donor cells. Approximate 4 × 10^7^ mixed donor cells were transplanted via IV injection into 4-week-old CD45.1^+^CD45.2^+^ heterozygous recipient mice including one male mouse and one female mouse that received two separate irradiation doses of 450 cGy. The reconstitution for WT vs Cd36-KO cells in the recipient mice was assessed by flow cytometry after 30 days of transplantation.

### Assessment of AML engraftment and progression in Cd36-KO mice

To assess the impact of *Cd36* deletion within the immune microenvironment and bone marrow niche on AML engraftment, we leveraged the FLT3-ITD/MLL-PTD mouse model, which is an aggressive leukemia model with 100% chance for recipient immune competent mice to develop leukemia [24]. 5 × 10^6^ FLT3-ITD/MLL-PTD mouse leukemic cells were separately intravenously injected into randomly selected 6- to 8-week-old CD45.2^+^ Cd36-KO mice (two males and three females) and CD45.2^+^ WT mice (two males and four females). Once the signs of disease burdens appeared, mice were euthanized and the bone marrow, spleen, liver, and blood were collected for flow cytometry analysis to identify the leukemia progression. In our AML engraftment study, the leukemic cells from CD45.1 hosts were dual expressing both CD45.1 and CD45.2, and all the Cd36-KO and WT recipient mice were uniformly positive for only CD45.2. The AML engraftment experiment was also repeated in a less aggressive mice model with reduced engrafted mouse leukemic cells, in which 1 × 10^6^ FLT3-ITD/MLL-PTD mouse leukemic cells were separately intravenously injected into randomly selected 3- to 4-month-old four male CD45.2^+^ Cd36-KO mice and four male CD45.2^+^ WT mice.

### Survival experiment

The survival experiment was performed to study the effects of *Cd36* knockout on overall survival (OS) between WT and Cd36-KO mice post transplantation with leukemic cells. Twenty-nine mice were allocated into four groups including seven male WT mice, five Cd36-KO male mice, seven WT female mice, and ten Cd36-KO female mice. About 1 × 10^6^ FLT3-ITD/MLL-PTD mouse leukemic cells were injected into 1- to 4-month-old mice through tail vein. The leukemia burden at disease progression was observed by following symptoms such as swollen eyes, swollen cheek, hunchback, tiredness, extreme difficulty in movement, and bare response to stimulation, then the mice were humanely euthanized, and survival time was recorded. The survival experiment was also repeated using 3- to 4-month-old twelve female mice including seven WT mice and five Cd36-KO mice engrafted with same number of FLT3-ITD/MLL-PTD mouse leukemic cells in a blinded manner.

### Statistical analysis

One-way ANOVA (Kruskal–Wallis test) was performed to compare the median difference of CD36 expression in normal human and mouse hematopoietic systems among each cell type. The significance of the differences between WT versus Cd36-KO groups were assessed by Mann–Whitney test, unpaired t-test, Welch's t-test, or ratio paired t-test in this study. The Kaplan–Meier and log-rank tests were used in survival study to analyze mice survival distribution by comparing median survival time (MST) with hazard ratio and 95% confidence interval (CI). The analysis results with a P value of less than 0.05 were considered significant. Data were presented as mean ± standard error of mean.

## Results

### *CD36* differential gene expression patterns in normal hematopoiesis between mouse and human

**Fig. 1:**
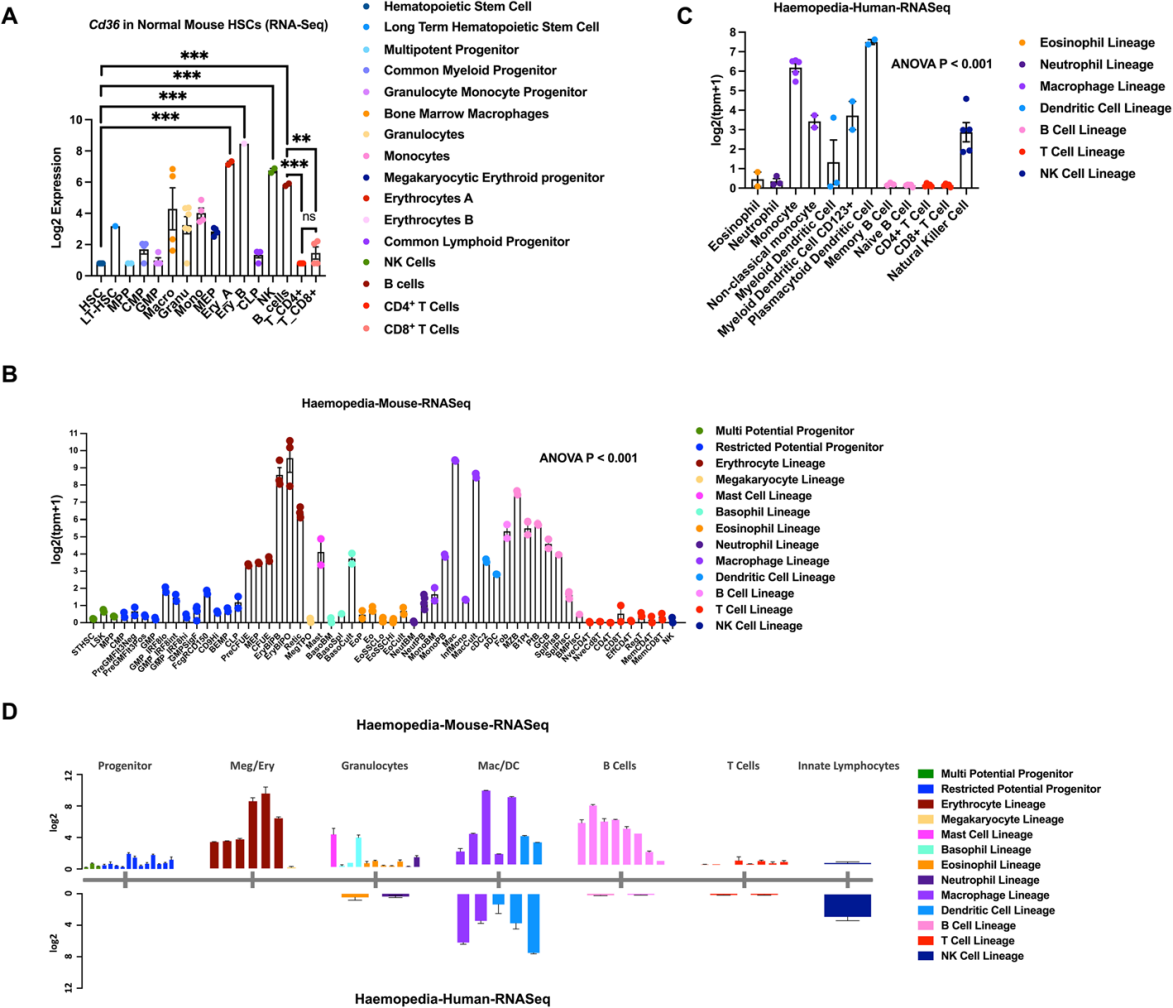
*Cd36* differential gene expression patterns in normal hematopoiesis between mouse and human. **A** Log2 transformed gene expression level for *Cd36* in normal mouse hematopoietic cells obtained from GSE60101 dataset downloaded from BloodSpot database. Data are presented as the mean of *Cd36* gene expression among each cell population and each colored dot represents the expression value of a single sample. Unpaired t-test analysis was used (***, P < 0.001; **, P < 0.01; ns, not significant). **B** Log2 transformed gene expression level for *Cd36* in normal mouse hematopoietic cells obtained from GSE116177 dataset downloaded from Haemosphere database. Data are presented as the mean of *Cd36* gene expression among each cell lineage and each colored dot represents the expression value of a single sample. One-way ANOVA test (Kruskal–Wallis test) was used for statistical analysis. **C** *CD36* expression in normal human hematopoietic cells obtained from GSE115736 dataset downloaded from Haemosphere database. Data are presented as the mean of *CD36* gene expression among each cell lineage and each colored dot represents the expression value of a single sample. One-way ANOVA test (Kruskal–Wallis test) was used for statistical analysis. **D** The comparison of RNA-Seq data between mouse and human for *Cd36* gene expression level. Data are presented as the mean of *Cd36* gene expression with standard error among each cell lineage

Multipotent long-term hematopoietic stem cells (LT-HSCs) reside in the bone marrow where they either self-renew and maintain the stem cell pool or differentiate into short-term HSCs (ST-HSCs) and lineage-restricted progenitors. These progenitors undergo extensive proliferation and differentiation, ultimately producing fully differentiated mature hematopoietic cells with specific functions [25–27]. To evaluate the expression patterns of *CD36* across the different hematopoietic stem cells, progenitors, and mature blood cells, we analyzed the GSE60101 mouse normal RNA-Seq dataset [15], downloaded from the public BloodSpot database. We found that in mouse normal hematopoiesis, *Cd36* expression was low in the hematopoietic stem cells (HSCs) and was gradually increased as cells differentiated into mature cells and particularly elevated in the erythrocytes A (115.4-fold, P < 0.001) and erythrocytes B (203.7-fold, P < 0.001) in the myeloid lineage, and NK cells (61.8-fold, P < 0.001) and B cells (32.9-fold, P < 0.001) in the lymphoid lineage (Fig. 1A). We also observed that *Cd36* expression is higher in B cells compared with CD4^+^ T cells (32.8-fold, P < 0.001) and CD8^+^ T cells (20.6-fold, P < 0.01) (Fig. 1A). Similar observations were seen when we analyzed another GSE116177 haemopedia mouse RNAseq data in the different hematopoietic lineages [13]. We observed higher level of *Cd36* expression in mature cells such as erythrocytes, mast cells, basophils, macrophages, dendritic cells, B cells, and T cells compared with hematopoietic progenitor cells (ANOVA P < 0.001, Fig. 1B). These results were validated in two additional datasets GSE14833 [16] and GSE6506 [17, 18] from BloodSpot. We found relatively low expression of *Cd36* in LT-HSCs, and elevated *Cd36* expression in erythrocyte lineage compared with the level of *Cd36* in LT-HSCs, particularly in the nucleated erythrocytes (87.4-fold, P < 0.001), 9.8-fold higher in erythroid progenitor cells (P < 0.001), and 2.8-fold higher in colony-forming unit erythroid cells (P < 0.05) (Additional file 1: Fig S1A). Higher expression level of *Cd36* mRNA than LT-HSCs was also observed in monocyte (7.4-fold, P < 0.01), B cell (7.5-fold, P < 0.001), and naïve CD8 positive T cells (1.6-fold, P < 0.05) (Additional file 1: Fig S1A). *Cd36* levels were higher in B cells compared with T cells subtypes including CD4^+^ T cells (7.3-fold, P < 0.01), activated CD4^+^ T cells (7.4-fold, P < 0.01), naïve CD8^+^ T cells (4.7-fold, P < 0.05), and activated CD8^+^ T cells (8.8-fold, P < 0.01) (Additional file 1: Fig S1A).

In normal human hematopoiesis, *CD36* mRNA was detected in B cell lineage, dendritic cell lineage, eosinophil lineage, macrophage lineage, neutrophil lineage, NK cell lineage, and T cell lineage (GSE115736 haemopedia, ANOVA P < 0.001, Fig. 1C) [13]. Relatively higher expression of *CD36* was found in dendritic cell lineage including myeloid dendritic cell, CD123^+^ myeloid dendritic cell, and plasmacytoid dendritic cell, monocytes, and NK cells. While low expression of *CD36* gene was found in memory B cell, naïve B cell, eosinophil, neutrophil, CD4^+^ T cell and CD8^+^ T cell. Additional datasets GSE17054 [19], GSE19599 [20], GSE11864 [21], and E-MEXP-1242 [22] were used for validation analysis. *CD36* expression level was significantly higher in monocytes with 158.3-fold (P < 0.001), in myeloid dendritic cells with 32.4-fold (P < 0.001), in plasmacytoid dendritic cells 80.8-fold (P < 0.001), and in CD4^+^ T cells with 12.6-fold (P < 0.001) compared with that in HSCs (Additional file 1: Fig S1B). Also, lower expression of *CD36* was found in B cells compared to CD4^+^ T cells (12.5-fold, P < 0.01) (Additional file 1: Fig S1B). Meanwhile, we identified higher *CD36* expression level in some restricted potential progenitor cells such as common myeloid progenitor cell (4.7-fold, P < 0.001), granulocyte monocyte progenitors (4.7-fold, P < 0.001), and megakaryocyte erythroid progenitor cell (69.9-fold, P < 0.001) compared with HSCs (Additional file 1: Fig S1B).

By comparing the RNA-Seq results from human and mouse, we observed differential *CD36* expression patterns between the two species. In mouse, *Cd36* gene expression was elevated in multi and restricted potential progenitors, erythrocytes, mast cell, and basophils in mouse. However, human *CD36* expression in multi and restricted potential progenitors and erythrocytes were not availbale from haemosphere dataset, meanwhile, *CD36* has higher relative expression levels to that in other lineages in human NK cells than in mouse NK cells (Fig. 1D). Also, *Cd36* expression levels in B cells were found relatively high in the mouse but reduced in human (Fig. 1D). On the other hand, macrophages, dendritic cells, and T cells showed similar patterns of *CD36* expression in both mouse and human (Fig. 1D).

### Cd36 knockout is confirmed in B6.129S1-*Cd36*^*tm1Mfe*^/J mouse tissues

*Cd36* knockout B6.129S1-*Cd36*^*tm1Mfe*^/J mice (Cd36-KO) were obtained from Jackson laboratory [23]. Mice genotypes were confirmed using recommended primers (Additional file 1: Fig S2A). *Cd36* knockout in CD36-KO mice was confirmed by assessing Cd36 surface expression levels with that in C57BL/6 J wildtype (WT) mice in the bone marrow (BM), spleen, liver, and blood by flow cytometry (Additional file 1: Fig S2E-H). Cd36^+^ population was reduced by 90–95% in Cd36-KO tissues compared with WT tissues (P < 0.0001, Additional file 1: Fig S2L-O). *Cd36* mRNA expression was also significantly reduced in BM tissues, spleens and livers of Cd36-KO mice compared with WT mice (P < 0.01, Additional file 1: Fig S2B-D). The spleen and liver of Cd36-KO mice looked normal and similar to organs obtained from the WT mice (Additional file 1: Fig S2I). The weights of spleen tissues were not significantly different between WT and Cd36-KO mice (WT vs Cd36-KO: 0.09 vs 0.08 g, P = 0.234; Additional file 1: Fig S2J), while the livers weighed slightly more in the Cd36-KO mice compared with the WT mice (WT vs Cd36-KO: 1.54 vs 1.79 g, P = 0.008; Additional file 1: Fig S2K).

### Cd36-KO mice exhibit minimal effect on blood count

To assess the effect of *CD36* knockout on normal hematopoietic cells, we performed a complete blood count (CBC) and differential blood analysis on blood samples from Cd36-KO mice and compared it with blood of WT mice (n = 6 mice per group). Hematological analysis including white blood cell (WBC) count, red blood cell (RBC) count, hemoglobin (Hb), hematocrit (PCV), mean corpuscular volume (MCV), mean corpuscular hemoglobin (MCH), mean corpuscular hemoglobin concentration (MCHC), neutrophil, lymphocytes, monocytes, eosinophils, basophils, and platelet counts were compared in Table 1. We noticed that compared to the blood from WT mice, the Cd36-KO blood exhibited slightly lower but still within the normal range of RBC count (WT vs Cd36-KO: 6.93 vs 5.85, P = 0.033, Additional file 1: Fig S3B), hemoglobin levels (WT vs Cd36-KO: 11.35 vs 10.03, P = 0.011, Additional file 1: Fig S3C), and hematocrit levels (WT vs Cd36-KO: 35.48 vs 31.67, P = 0.020, Additional file 1: Fig S3D). There was no significant difference for white blood cell count (WT vs Cd36-KO: 2.06 vs 1.47, P = 0.246, Additional file 1: Fig S3A), neutrophil percentage (WT vs Cd36-KO: 6.10 vs 5.77, P = 0.875, Additional file 1: Fig S3E), lymphocytes percentage (WT vs Cd36-KO: 86.48 vs 81.55, P = 0.151, Additional file 1: Fig S3F), and platelet count (WT vs Cd36-KO: 280.17 vs 230.67, P = 0.678, Additional file 1: Fig S3H) in Cd36-KO mice compared with WT mice. Cd36-KO mice exhibit relatively higher but not statistically significant monocytes percentage (WT vs Cd36-KO = 3.23 vs 5.27, P = 0.085, Additional file 1: Fig S3G) than WT mice. Overall, the hematology analysis results revealed similar blood phenotype between both Cd36-KO and WT mice.

**Table 1:** Hematology analysis between Cd36-KO and WT mice blood

	WT (Mean ± SD)	Cd36-KO (Mean ± SD)	Reference range	P value (T test)
White blood cell (× 10^3^/μL)	2.06 ± 0.90	1.47 ± 0.76	4.8–10.8	0.246
Red blood cell (× 10^6^/μL)	6.93 ± 0.87	5.85 ± 0.63	4.7–6.1	0.033
Hemoglobin (g/dL)	11.35 ± 0.84	10.03 ± 0.61		0.011
Hematocrit (%)	35.48 ± 2.16	31.67 ± 2.60		0.020
Mean corpuscular volume (fL)	51.70 ± 4.97	54.62 ± 6.49	80–100	0.403
Mean corpuscular hemoglobin (pg)	16.50 ± 1.43	17.32 ± 1.70	26–34	0.389
Mean corpuscular Hemoglobin concentration (%)	31.98 ± 0.61	31.75 ± 1.17	31–36	0.675
Neutrophil (%)	6.10 ± 4.59	5.77 ± 2.14		0.875
Lymphocytes (%)	86.48 ± 5.83	81.55 ± 5.13		0.151
Monocytes (%)	3.23 ± 1.33	5.27 ± 2.24		0.085
Eosinophils (%)	4.08 ± 4.55	7.40 ± 6.33		0.322
Basophils (%)	0.10 ± 0.17	0.02 ± 0.04		0.263
Platelet (Autom) (× 1000//μL)	280.17 ± 231.47	230.67 ± 163.59	150–40	0.678
Neutrophil absolute	114.33 ± 89.73	85.67 ± 63.65		0.538
Lymphocytes/absolute	1780.50 ± 735.10	1201.17 ± 632.25		0.174
Monocytes absolute	69.50 ± 44.36	86.50 ± 72.46		0.635
Eosinophils absolute	97.00 ± 158.15	98.00 ± 79.46		0.989
Basophils absolute	1.50 ± 2.35	0.17 ± 0.41		0.200

### T cells from Cd36-KO mice exhibit similar phenotypes and in vitro expansion compared with that of WT mice

**Fig. 2:**
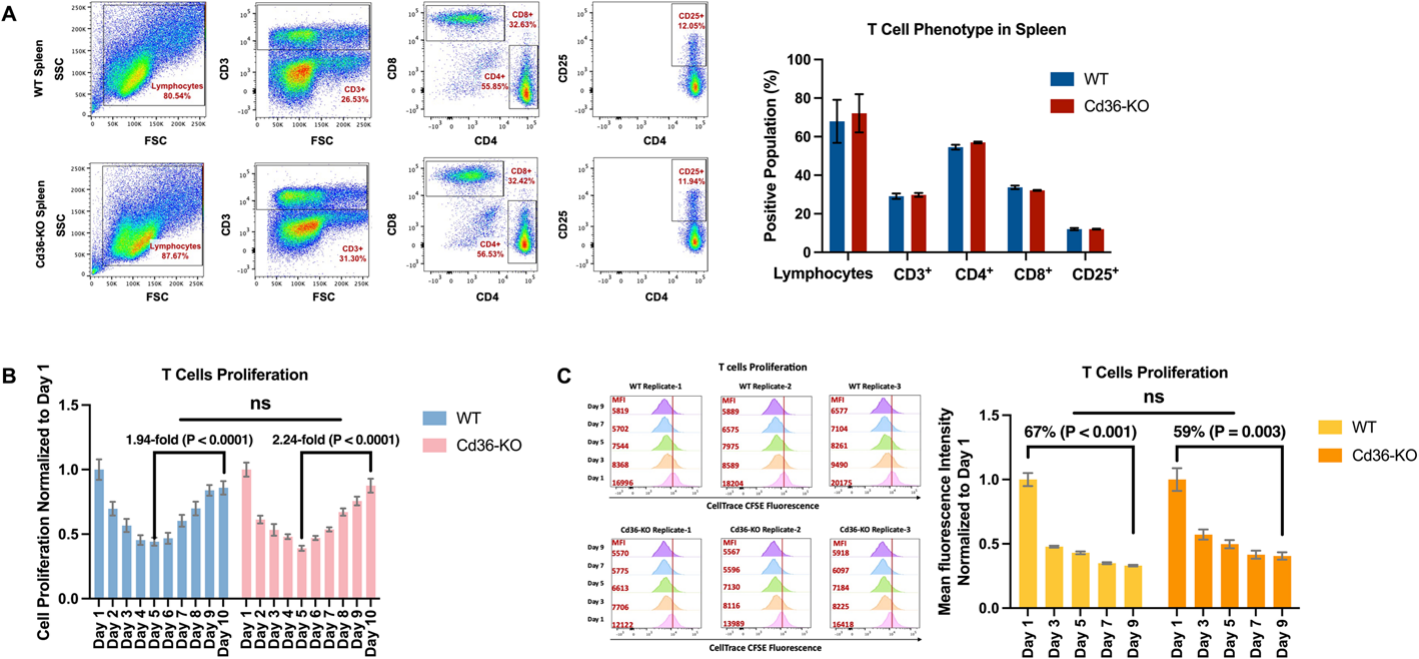
Cd36-KO mice exhibit similar T cell phenotypes and in vitro expansion compared with WT mice. **A** Representative flow cytometry of the lymphocytes population from mouse spleen cells stained with CD3, CD4, CD8, and CD25 flow antibody to evaluate the different T cell population in WT mice and Cd36-KO mice. Quantification results of the positive cell population percentages were represented by the bar graph, in which each bar represents the mean with standard error of population percentage for Cd36-KO and WT mice (n = 6 mice per group). **B** A continuous 10-day proliferation assay was performed on healthy mouse primary spleen cells (n = 3 biological replicates). The cell numbers among WT and Cd36-KO groups were normalized to the first day and then were compared. Unpaired t-test was used to analyze the fold change difference. (ns, not significant). **C** The flow cytometry analysis demonstrated that CellTrace CFSE fluorescent stains from permanently labeled parent cells were diluted through subsequent cell divisions, and reduced fluorescent intensities were similar in daughter cells between WT and Cd36-KO mice spleen cells (n = 3 biological replicates). The bar graph showed similar T cell expansion in splenocytes between WT and Cd36-KO mice by comparing the mean of fluorescence intensity with standard error on day 1, 3, 5, 7, and 9. Unpaired t-test was used to analyze the percentage difference (ns, not significant)

Considering the increased interest in the role of Cd36 in T cells and cancer immune microenvironment, and the similarity in *CD36* expression patterns in T cells between human and mouse, we next assessed T cell phenotypes in Cd36-KO mice. We compared the T cell populations based on the expression of T cell surface markers including CD3, CD4, CD8 and CD25 in the lymphocytes of spleen tissue between Cd36-KO and WT mice (n = 6 mice per group). We observed that Cd36-KO mice exhibited similar T cell phenotype to that in the WT mice in spleen cells by comparing the positive T cell populations for lymphocytes (WT vs Cd36-KO: 67.97 ± 27.38% vs 72.13 ± 24.21%; P = 0.786), CD3^+^ (WT vs Cd36-KO: 29.10 ± 3.50% vs 29.80 ± 2.49%; P = 0.698), CD4^+^ (WT vs Cd36-KO: 54.52 ± 3.04% vs 57.02 ± 1.18%; P = 0.090), CD8^+^ (WT vs Cd36-KO = 33.70 ± 2.21% vs 32.12 ± 0.68%; P = 0.125), and CD25^+^ (WT vs Cd36-KO: 12.03 ± 1.49% vs 12.03 ± 0.55%; P = 0.992) (Fig. 2A, Additional file 1: Fig S4).

To assess the impact of CD36-KO on T cell in vitro expansion, proliferation assay was performed on mouse splenocytes for 10 days in media supplemented with interleukin-2 (IL-2) cytokine and phytohemagglutinin (PHA). Daily cell count showed that expansion of T cells started on the 5^th^ day of cell culture in both WT splenocytes and Cd36-KO splenocytes (Fig. 2B). Also, the WT splenocytes showed 1.94-fold (P < 0.0001) expansion of T cells on day 10 relative to day 5, while the expansion rate was 2.24-fold (P < 0.0001) in Cd36-KO splenocytes (P =  0.252 between WT and Cd36-KO, Fig. 2B). The proliferated T cells in WT and Cd36-KO splenocytes were also confirmed by tracking the CellTrace CFSE dye by flow cytometry, which was represented by the diluted fluorescence intensity through subsequent T cell divisions on day 1, day 3, day 5, day 7, and day 9 (Fig. 2C). It was found that WT splenocytes had 67% (P < 0.001) reduced mean fluorescence intensity on day 9 relative to day 1, and the Cd36-KO splenocytes had 59% (P = 0.003) reduction (P = 0.288 between WT and Cd36-KO, Fig. 2C).

### Loss of Cd36 is associated with reduced hematopoietic stem and progenitor cells colony formation

**Fig. 3:**
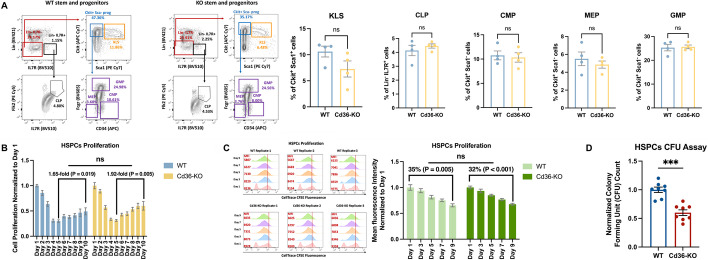
Loss of *Cd36* is associated with reduced hematopoietic stem and progenitor cells colony formation. **A** Representative flow cytometry result showing the stem and progenitors as a percentage of ckit and Il7rα fresh and enriched BM cells collected from both WT mice and CD36-KO mice (n = 4 female mice per group); early form of murine hematopoietic stem cell (KLS), common lymphoid progenitor (CLP), common myeloid progenitor (CMP), megakaryocyte-erythroid progenitor (MEP), and granulocyte–macrophage progenitor (GMP). Quantification results of the positive cell population percentages were represented by the bar graph, in which each bar represents the mean with standard error of population percentage for Cd36-KO and WT mice (n = 4 mice per group). The differences between groups were analyzed using unpaired t-test (ns, not significant). **B** A continuous 10-day proliferation assay was performed on BM cells (n = 3 biological replicates). The cell numbers among WT and Cd36-KO groups were normalized to the first day and then were compared. Unpaired t-test was used to analyze the fold change difference. (ns, not significant). **C** Flow cytometry analysis demonstrated that CellTrace CFSE fluorescent stains from permanently labeled parent cells were diluted through subsequent cell divisions, and the reduced fluorescent intensities were similar in daughter cells between WT and Cd36-KO mice BM cells (n = 3 biological replicates). The bar graph showed similar HSPCs expansion in BM between WT mice and Cd36-KO mice by comparing the mean of fluorescence intensity with standard error on day 1, 3, 5, 7, and 9. Unpaired t-test was used to analyze the percentage difference. (*ns*, not significant). **D** The colony forming units (CFUs) were manually counted for each replicate in both Cd36-KO and WT groups, and then normalized relative to the mean counts of WT group. The colored bar graph represents mean with standard error of CFU count for WT and Cd36-KO mice group. Each colored dot represents the count of each single replicate (n = 8 biological replicates). The difference between WT and Cd36-KO group was analyzed by Mann–Whitney test (***, P < 0.001)

To examine the impact of *Cd36* deletion on normal hematopoiesis, we assessed the percentage of the different hematopoietic stem and progenitor populations in the enriched BM cells collected from female Cd36-KO mice in comparison with female WT mice. We found no significant difference when comparing the population of the early form of murine hematopoietic stem cells (KLS), common lymphoid progenitors (CLPs), common myeloid progenitor (CMP), megakaryocytic-erythroid progenitors (MEP), and granulocyte-monocyte progenitor (GMP) between Cd36-KO and WT mice (Fig. 3A). Similar observation was found in both unenriched-fresh BM cells collected from female Cd36-KO and WT mice (Additional file 1: Fig S5A) and unenriched-frozen BM cells obtained from male Cd36-KO and WT mice (Additional file 1: Fig S5B).

Mice bone marrow cells were cultured in 20% FBS-RPMI supplemented with stem cell factor (SCF) and thrombopoietin (TPO) cytokines for hematopoietic stem and progenitor cells (HSPC) expansion over 10 days in vitro. Both HSPCs obtained from the Cd36-KO and WT mice showed similar rates of in vitro expansion starting on 5th day of cell culture (day 10 relative to day 5 change in cell number: 1.65-fold (P = 0.019) in HSPCs from WT mice and 1.92-fold (P = 0.005) in HSPCs from Cd36-KO mice, P =  0.246 between WT and Cd36-KO, Fig. 3B). HSPCs proliferation evaluated by monitoring CellTrace carboxyfluorescein succinimidyl ester (CFSE) dye dilution further confirmed a similar growth pattern between WT and Cd36-KO BM cells (Fig. 3C). The mean fluorescence intensity was decreased by 35% (P = 0.005) in WT HSPCs and 32% (P < 0.001) in Cd36-KO HSPCs on day 9 relative to day 1 (P = 0.829 between WT and Cd36-KO, Fig. 3C).

To investigate the colony forming ability of HSPCs, bone marrow cells from Cd36-KO and WT mice (n = 3 per group) were cultured in a semi-solid methylcellulose based medium in response to cytokine stimulation. The colonies were counted on day 8 after plating. Cd36-KO BM cell resulted in 40% fewer colonies than WT mouse (P < 0.001, Fig. 3D).

### Cd36 is dispensable for normal HSPCs homing and engraftment in the bone marrow

**Fig. 4:**
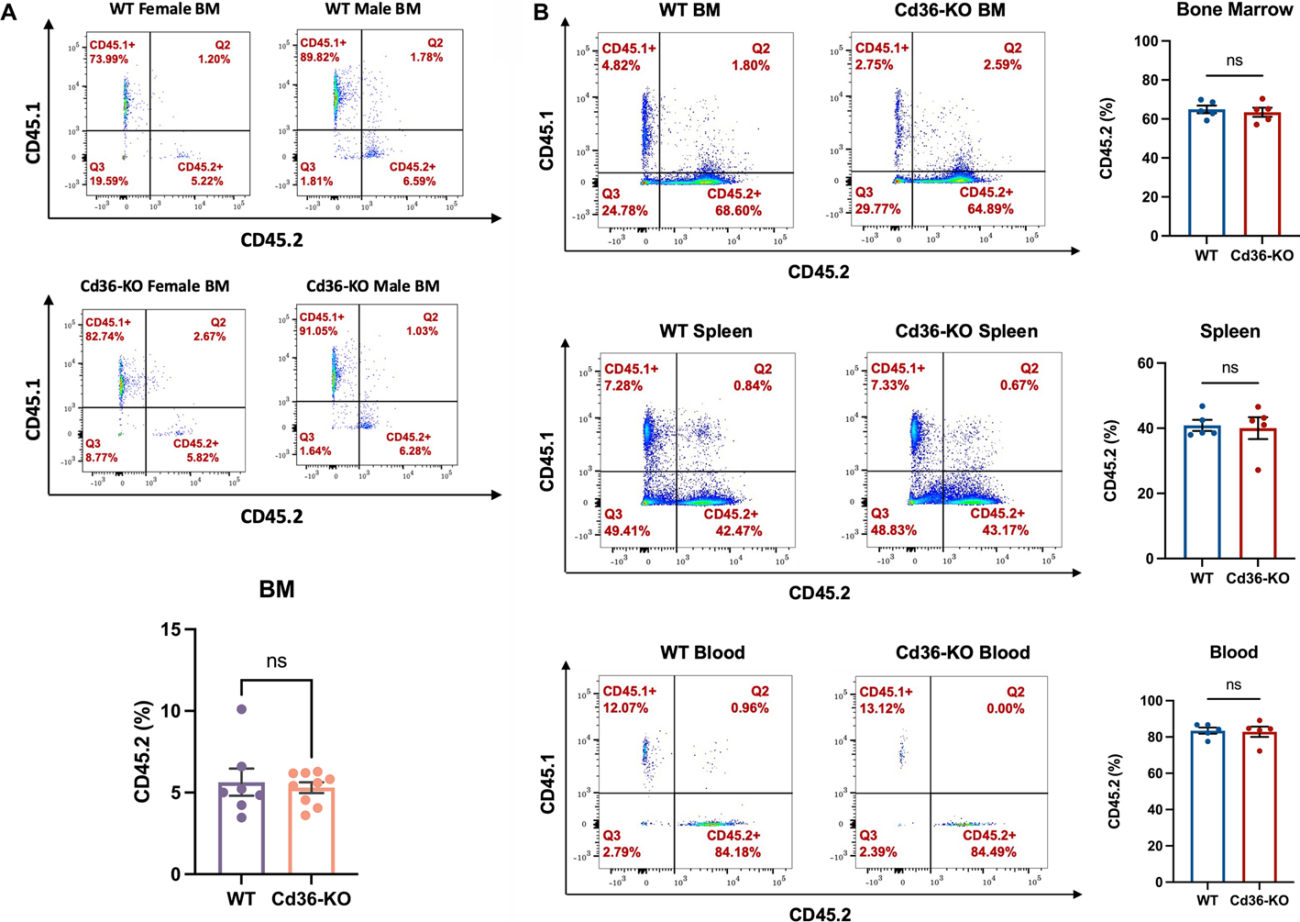
*Cd36* is dispensable for normal HSPCs homing and engraftment in the bone marrow. **A** Representative flow cytometry results showing CD45.2^+^ WT or Cd36-KO HSPCs homing in CD45.1^+^ recipient BM niches (n = 7 mice in WT group and n = 9 mice in Cd36-KO group). The difference between groups was analyzed using Mann–Whitney test (ns, not significant). **B** Representative flow cytometry results showing BM transplant and quantification of engraftment of healthy CD45.2^+^ WT or CD36-KO BM cells in the BM, spleen, and blood cells of CD45.1 allele bearing mice (n = 5 mice per group). The differences between groups were analyzed using Mann–Whitney test (ns, not significant)

To examine the effect of *Cd36* knockout on the homing of HSPCs to the bone marrow, we transplanted 2.0–2.4 × 10^7^ BM cells obtained from the femur of either WT or Cd36-KO mice carrying CD45.2^+^ allele into irradiated CD45.1^+^ mice. Approximately, 15 hrs later, recipient mice were sacrificed, and BM cells were isolated and analyzed by flow cytometry to determine the percentage of CD45.2^+^ homing HSPCs. We found similar percentage of homing to the BM in both groups: 5.64% for WT HSPCs and was 5.30% for Cd36-KO HSPCs (P = 0.837, Fig. 4A, Additional file 1: Fig S6).

Next, we asked whether deletion of the *Cd36* gene affects the long term engraftment and expansion of HSPCs in recipient mice. We transplanted 2.4 × 10^7^ healthy mouse BM cells collected from CD45.2 allele bearing WT mice or CD36-KO mice into CD45.1 recipient mice (n = 5 mice per group). Recipient mice were sacrificed 35 days post-transplant. The weight of spleens and livers were not significantly different between groups (Additional file 1: Fig S7A–C). Compared with the BM cells from the WT mice, Cd36-KO cells showed similar engraftment (% CD45.2^+^ engraftment means in BM, 64.92 vs 63.45, for WT vs Cd36-KO, P > 0.999, Fig. 4B, Additional file 1: Fig S7D; % CD45.2^+^ engraftment means in spleen, 40.88 vs 40.05, for WT vs Cd36-KO, P = 0.841, Fig. 4B, Additional file 1: Fig S7E; % CD45.2^+^ engraftment means in blood, 83.47 vs 82.88, for WT vs Cd36-KO, P > 0.999, Fig. 4B, Additional file 1: Fig S7F). The results indicated that *Cd36* expression is not required for the transplantation of normal HSPCs.

We also performed the competitive repopulation assay to assess the HSPCs reconstitution ability in irradiated CD45.1^+^CD45.2^+^ mice, we transplanted 4 × 10^7^ mixed CD45.1^+^ WT BM cells and CD45.2^+^ Cd36-KO BM cells at 1:1 ratio into each 4-week-old irritated one male and one female mice. The HSPCs reconstitution in BM and spleen was analyzed by flow cytometry after 30 days of transplantation. We observed that the CD45.1^+^ WT cells were significantly higher than the CD45.2^+^ Cd36-KO cells in the recipient BM cells (% engraftment means, 25.75 vs 17.53, for WT vs Cd36-KO, P = 0.025, Additional file 1: Fig S8A) and spleen cells (% engraftment means, 24.12 vs 8.98, for WT vs Cd36-KO, P = 0.015, Additional file 1: Fig S8B). The engraftment of CD45.1^+^ WT cells versus CD45.2^+^ Cd36-KO cells in the recipient blood cells had no significant difference (% engraftment means, 0.215 vs 0.115, for WT vs Cd36-KO, P = 0.305, Additional file 1: Fig S8C). While this analysis is limited to two mice and the transplanted cells are from CD45.1 and CD45.2 mice, it may suggest that the Cd36-KO BM cells were less competitive than WT-BM cells in terms of reconstitution ability.

### Deletion of *CD36* in the microenvironment has no effect on leukemia progression

**Fig. 5:**
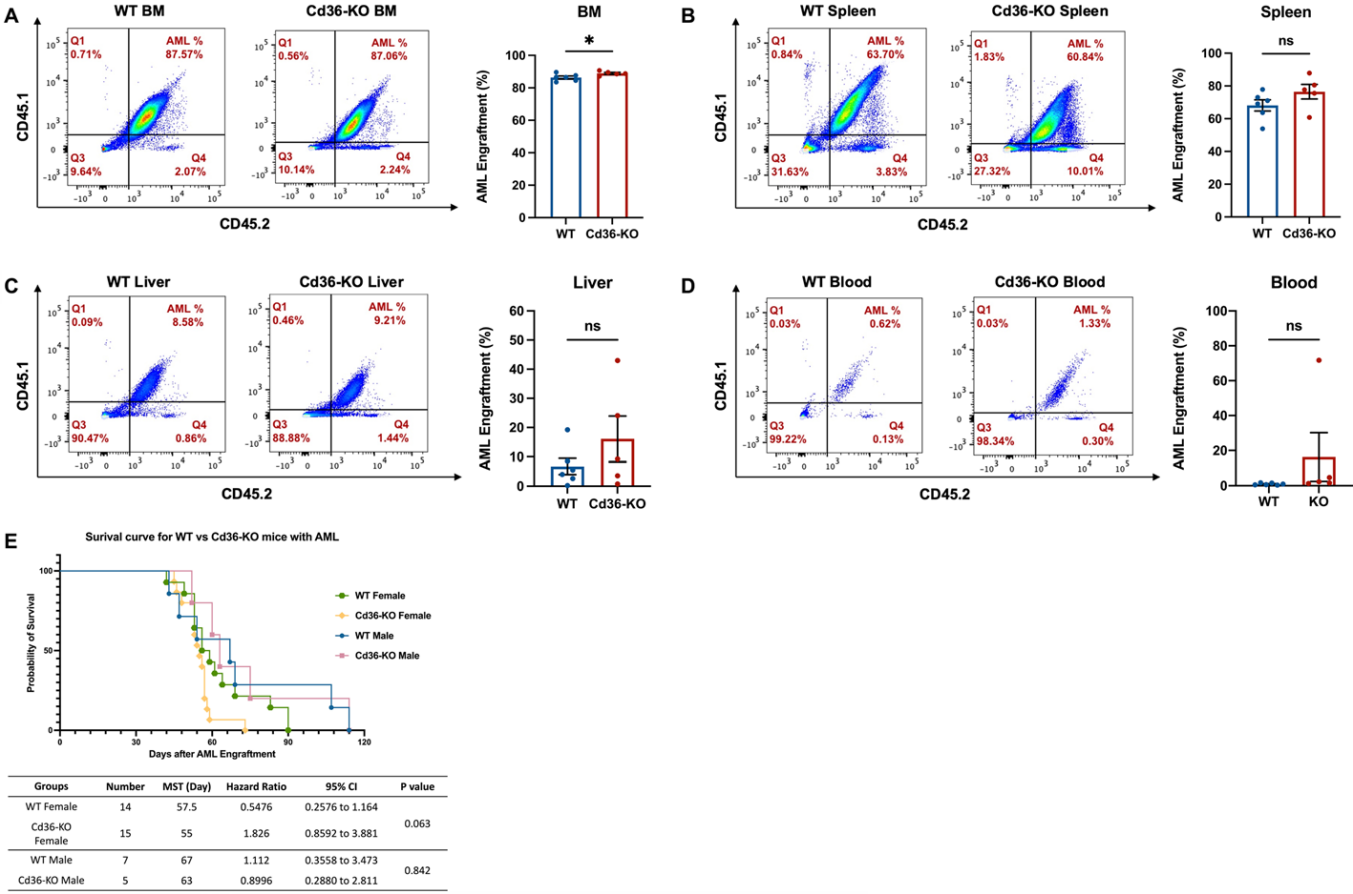
Cd36-KO mice exhibit similar AML engraftment compared with WT mice. **A**–**D** Representative flow cytometry result showing AML engraftment and quantification of engraftment of FLT3-ITD/MLL-PTD mouse leukemic cells in the BM, spleen, blood, and liver cells of WT and Cd36-KO mice (n = 6 mice in WT group; n = 5 mice in Cd36-KO group). The differences between groups were analyzed using unpaired t test (*, P < 0.05; *ns* not significant). **E** Kaplan–Meier survival curve of WT mice and Cd36-KO mice. (Log-rank test for survival distribution among WT female vs Cd36-KO female and WT male vs Cd36-KO male, respectively)

To examine whether *Cd36* knockout in the mouse microenvironment affects leukemia progression, we engrafted 5 × 10^6^ FLT3-ITD/MLL-PTD mouse leukemic cells by intravenous tail injection into Cd36-KO and WT mice to monitor the AML development (n = 5 in CD36-KO group, n = 6 in WT group). The mice were sacrificed between day 20 – 24 after observing the disease symptoms. The bone marrows, spleens and livers of Cd36-KO and WT mice were collected (Additional file 1: Fig S9A) and evaluated for their weight and leukemia engraftment. We found no significant difference in the weight of spleens (WT vs Cd36-KO = 0.24 vs 0.16 g, P = 0.082; Additional file 1: Fig S9B) or livers (WT vs Cd36-KO = 1.38 vs 1.48 g, P = 0.662; Additional file 1: Fig S9C) between Cd36-KO and WT. Mice from the Cd36-KO group exhibited slightly higher leukemia engraftment in the BM, but not in the spleen, liver, and blood compared with the WT group (% BM engraftment means, 88.92 vs 86.38, for Cd36-KO vs WT, P < 0.05, Fig. 5A, Additional file 1: Fig S9D; % spleen engraftment means, 76.48 vs 68.10, for Cd36-KO vs WT, P = 0.163, Fig. 5B, Additional file 1: Fig S9E; % liver engraftment means, 16.12 vs 6.70, for Cd36-KO vs WT, P = 0.252, Fig. 5C, Additional file 1: Fig S9F; % blood engraftment means, 16.33 vs 0.96, for Cd36-KO vs WT, P = 0.250, Fig. 5D, the large difference is due to one outlier mouse with high engraftment, Additional file 1: Fig S9G).

To address whether *Cd36* knockout may have an impact on the leukemia progression when less aggressive models is used, 1 × 10^6^ FLT3-ITD/MLL-PTD mouse leukemic cells were intravenously injected into Cd36-KO and WT mice and mice were sacrificed after 30 days later. The bone marrows, spleens and livers of Cd36-KO and WT mice were collected and analyzed (Additional file 1: Fig S10A). Similar with the previous model, no significant difference in the weight of spleens (WT vs Cd36-KO = 0.27 vs 0.23 g, P = 0.886; Additional file 1: Fig S10B) and livers (WT vs Cd36-KO = 1.96 vs 2.11 g, P = 0.114; Additional file 1: Fig S10C) between Cd36-KO and WT. The leukemia burden in the BM, spleen, liver, and blood were also similar between Cd36-KO and WT mice (% BM engraftment means, 90.66 vs 85.89, for Cd36-KO vs WT, P = 0.232, Additional file 1: Fig S10D; % spleen engraftment means, 39.87 vs 53.18, for Cd36-KO vs WT, P = 0.249, Additional file 1: Fig S10E; % liver engraftment means, 16.94 vs 26.34, for Cd36-KO vs WT, P = 0.606, Additional file 1: Fig S10F; % blood engraftment means, 41.36 vs 49.71, for Cd36-KO vs WT, P = 0.766, Additional file 1: Fig S10G).

In addition, we performed survival analysis to address whether the deletion of *Cd36* in recipient mice influence leukemia progression. In both female and male mice, the survival of the Cd36-KO mice where not significantly different from the WT mice when each group were engrafted with FLT3-ITD/MLL-PTD cells (female: median survival for WT versus Cd36-KO: 57.5 vs 55 days (P = 0.063, Fig. 5E; male: median survival for WT versus Cd36-KO: 67 vs 63 days (P = 0.842, Fig. 5E).

## Discussion

As a fatty acid receptor, CD36 was found to play a major role in metastasis initiation and potentially a therapeutic target to impair metastasis [28, 29]. In AML, we have previously demonstrated that CD36 acts with the secreted protein APOC2 to drive leukemia progression in mouse models via activation of ERK signaling [11]. Additionally, CD36 was reported to facilitate the escape of the leukemic stem cells (LSCs) from chemotherapy by a unique metabolism mechanism. This study found that LSCs were segregated by two populations depending on the expression level of CD36 and CD36^+^ LSCs prefer homing to gonadal adipose tissue (GAT), which GAT lipolysis fueled fatty acid oxidation for LSCs and further provided the niche for CD36^+^ LSCs developing chemo-resistance [30]. However, *CD36* is widely expressed in a variety of adaptive and innate immune cells and participates in several cell signaling and metabolic pathways, ultimately impacting on immune cell differentiation and activation [31]. In monocytes, it was found that CD36 was devoted to the differentiation to macrophages [32] and dendritic cells [33]. CD36 plays important roles in macrophages, including phagocytosis of apoptotic cells [34], inducing inflammatory response [35], and foam cell formation [36]. Thus, evaluating the consequences of targeting CD36 on normal hematopoietic cells, will provide needed answers into whether CD36 presents a viable therapeutic target in cancer.

The availability of a *Cd36* knockout mouse model provides a unique opportunity to assess whether *CD36* deletion has a detrimental impact on normal hematopoietic system. Comparative analysis of patterns of *CD36* expression between mouse and human is needed to translate the finding from the mouse model to human. Analysis of several RNAseq data for mouse *Cd36* showed high *Cd36* expression in mouse erythrocytes. However, previous studies suggested undetectable or very low levels of CD36 on adult human RBCs [37]. Another earlier study suggested the absence of glycoprotein IV (GPIV, CD36) in healthy human platelets with negative platelet-specific antigen NaK^a^ [38]. Also, healthy blood donors did not exhibit apparent evidence of hemostatic problems, suggesting the deficiency of platelets *CD36* in NaK^a−^ subjects was not pathogenic [38]. Additionally, we found relatively higher *Cd36* expression in B cells from the normal mice hematopoietic system, and the highest *Cd36* expression was detected in marginal zone (MZ) B cells compared to other B cell subsets according to the previous finding [39]. The differential expression of CD36 on B cell subsets was found to modulate the antigen response during B cell development [39], and the *CD36*-deficient B cells exhibited impaired autophagosome formation, leading to less plasma cell production and attenuated T cell dependent immune responses [40]. Differently from the mouse data, RNA-Seq data suggested that *CD36* expression was scarcely detected in human B cells. Altogether, these differences between mouse and human *CD36* expression patterns should be considered when assessing the impact of *CD36* deletion or inhibition and translating these findings to human. Except for the common erythrogram evaluation parameters including RBC, Hb, Hct, and MCHC [41], showing a slightly decreased levels (but remain within the normal ranges) in the Cd36-KO mice blood compared with WT mice blood, our study shows normal blood phenotypes. Considering the lack/low *CD36* expression in human erythrocytes, it is possible that targeting CD36 may have limited if any impact on human RBCs.

A recent study identified a critical role of CD36 for HSC metabolism during acute infection, allowing the metabolic transition from glycolysis towards β-oxidation [42]. In the absence of stress conditions (such as infections or competition), our results showed that mouse HSPCs from Cd36-KO mice expanded normally both in vitro and in vivo. However, our study found that HSPCs from Cd36-KO mice resulted in developing fewer colonies in methylcellulose-based colony assay and slight reduction in the erythrocytes. Despite this, we observed that the populations of HSPCs were not significantly different between Cd36-KO BM and WT BM by analyzing the KLS, CLP, CMP, GMP, and MEP fractions. It is yet to be determined whether the decrease in colony formation observed in HSPCs from CD36-KO mice has any adverse effects on the hematopoietic system under stress conditions. Even though the engraftment of HPSCs was less with the loss of *Cd36* gene in the competitive repopulation assay, *Cd36* deficient HPSCs still exhibited functional hematopoiesis reconstitution ability to bone marrow when transplanted alone. Deletion of *Cd36* gene did not affect the homing or the engraftment of HSPCs in the non-competitive transplantation assays. The results demonstrated the successful transplantation of Cd36-KO HSPCs in the reconstitution of multilineage hematopoiesis in both the short and longer term.

Recent studies suggest that CD36 play a major role within the tumor immune microenvironment (TIME), CD36-mediated fatty acid uptake induced lipid peroxidation and ferroptosis, which impaired the cytotoxic CD8^+^ T cell function and reduced its anti-tumor activity [43]. Furthermore, the increased expression levels of CD36 enhances lipid accumulation, and oxidized fatty acid levels leading to the differentiation and activation of tumor-associated macrophages [44]. Considering the increased interest in how CD36 impact T cell lipid metabolism within the immune microenvironment, we analyzed whether *Cd36* deletion has an impact on T cell phenotypes and T cell proliferation. However, our studies showed no significant difference between the different T cell populations between Cd36-KO and WT mice. A recent study has shown that monocytes presenting higher CD36^+^ marker induce Foxp3^+^ and CD25^+^ T cells from CD4^+^ and CD8^+^ T cells population [45]. However, our results revealed similar % of CD25^+^ cells between Cd36-KO and WT spleen cells, and similar T cells expansion from both groups in the presence of IL-2 and PHA.

Furthermore, the deletion of *Cd36* in the leukemia microenvironment had little impact on leukemia progression. The engraftments of MLL-PTD/FLT3-ITD cells in Cd36-KO mice were not different from that in the WT mice. Whether this effect is unique to the leukemia model used in our studies remains to be explored.

## Conclusions

Our study indicated that *Cd36* was differentially expressed in the hematopoiesis between mouse and human. We also demonstrated that *Cd36* is dispensable for HSPCs expansion and engraftment and that the deletion of *Cd36* has minimal impact on normal hematopoietic cells and leukemia immune microenvironment. Overall, this study suggested that a therapeutic approach aimed at targeting Cd36 will have limited hematological toxicities. These findings will advance the development of therapeutic approaches targeting CD36 particularly in AML and broadly in cancer.

## Supplementary Information

**Additional file 1: Table S1.** *Cd36* Gene Expression in Mouse Normal Hematopoiesis. **Table S2.** Gene Expression for *Cd36* in Mouse Normal Hematopoiesis. **Table S3.** Gene Expression for *CD36* in Human Normal Hematopoiesis. **Table S4.** *Cd36* expression in mouse normal hematopoietic system. **Table S5**. *CD36* expression in normal human Hematopoiesis. **Figure S1.** *Cd36* differential gene expression patterns in normal hematopoiesis between mouse and human. (A-B) Log2 transformed gene expression level for *Cd36* in mouse normal hematopoietic system obtained from GSE14833 and GSE6506 datasets and *CD36* expression in normal human hematopoiesis obtained from datasets GSE17054, GSE19599, GSE11864, and E-MEXP-1242, which all datasets were downloaded from BloodSpot database. Data are presented as the mean of gene expression among each cell population and each colored dot represents the expression value of a single sample. Unpaired t-test analysis was used (***, P < 0.001; **, P < 0.01; *, P < 0.05). **Figure S2.** Confirmation of Cd36 reduced expression in Cd36-KO mice compared with WT mice. (A) Genotype confirmation of wildtype and *Cd36* knockout mice by gel electrophoresis of DNA fragments generated by standard PCR with recommended primers. Lane 1 and 2: WT mice liver tissues with WT primers; Lane 3 and 4: WT liver mice tissues with Cd36-KO primers; Lane 5 and 6: Cd36-KO liver mice tissues with WT primers; Lane 7 and 8: Cd36-KO mice tissues with Cd36-KO primers. (B-D) qPCR quantification of *Cd36* knockout efficiency in healthy mouse bone marrow cells, spleen cells, and liver cells (n = 6 mice per group). The bar graph represents the mean of *Cd36* mRNA level in per group with standard error of the mean. Welch's t-test was used to analyze the significant difference (**, P < 0.01). (E-H) Cd36 knockout was confirmed using flow cytometry by comparing the cell surface Cd36^+^ population in bone marrow, spleen, liver, and blood tissues (n = 6 mice per group). (I) The spleens and livers were collected from Cd36-KO and WT mice. (J-K) The weight of spleen and liver organs were compared. The bar graph represents the mean of spleen and liver mass in Cd36-KO and WT mice with standard error of the mean. Each colored dot represents the mass value of every single mouse tissue (n = 6 mice per group). The differences between Cd36-KO and WT mice tissues were analyzed by unpaired t-test (**, P < 0.01; Abbreviation: ns, not significant). (L-O) Cd36 knockout efficiency was confirmed using flow cytometry analysis by comparing the cell surface Cd36^+^ population and Cd36 MFI in BM, spleen, liver, and blood cells. Data are presented as the mean of Cd36^+^ surface expression or CD36 MFI and each colored dot represents the value of a single sample. (n = 6 mice per group). The differences between Cd36-KO and WT mice tissues were analyzed by Welch's t-test (*, P < 0.05; ***, P < 0.001; ****, P < 0.0001). **Figure S3.** Hematological analysis reveals similar blood counts between Cd36-KO and WT mice. (A-H) Data are presented as the mean of blood count between Cd36-KO mice and WT mice for white blood cell, red blood cell, hemoglobin, hematocrit, neutrophil, lymphocyte, monocyte, and platelet. Each colored single dot represents the count for every single mouse (n = 6 mice per group). The differences between Cd36-KO and WT group were analyzed by unpaired t-test (*, P < 0.05; Abbreviation: ns, not significant). **Figure S4.** Cd36-KO mice exhibit similar T cell phenotypes compared with WT mice. (A-B) Representative flow cytometry of the lymphocytes population from mouse spleen cells stained with CD3^+^, CD4^+^, CD8^+^, and CD25^+^ flow antibody to evaluate the different T cell population in WT mice and Cd36-KO mice (n = 6 mice per group). **Figure S5.** Chracterization of hematopoietic stem and progenitors in WT and Cd36-KO mice. (A) Quantification results of the positive cell population percentages in freshly unriched BM cells were represented by the bar graph, in which each bar represents the mean with standard error of population percentage for Cd36-KO and WT mice (n = 4 female mice per group). Shown here are an early form of murine hematopoietic stem cell (KLS), common lymphoid progenitor (CLP), common myeloid progenitor (CMP), megakaryocyte-erythroid progenitor (MEP), and granulocyte-macrophage progenitor (GMP). The differences between groups were analyzed using unpaired t-test (Abbreviation: ns, not significant). (B) Quantification results of the positive cell population percentages in frozen unriched BM cells were represented by the bar graph, in which each bar represents the mean with standard error of population percentage for Cd36-KO and WT mice (n = 2 male mice per group). Shown here are an early form of murine hematopoietic stem cell (KLS), common lymphoid progenitor (CLP), common myeloid progenitor (CMP), megakaryocyte-erythroid progenitor (MEP), and granulocyte-macrophage progenitor (GMP). The differences between groups were analyzed using unpaired t-test (Abbreviation: ns, not significant). **Figure S6.** The homing rate was similar between WT and Cd36-KO mice. Representative flow cytometry result showing CD45.2^+^ WT or Cd36-KO HSPCs homing in CD45.1^+^ recipient BM niches (n = 7 in WT including 5 female and 2 male in WT group and n = 9 in Cd36-KO including 5 female and 4 male). **Figure S7.** *Cd36* is dispensable for normal HSPCs homing and engraftment in the bone marrow. (A) Spleens and livers were collected from CD45.1 allele bearing mice injected with either 2.4 x 10^7^ CD45.2^+^ BM cells (WT; n = 5 mice) or 2.4 x 10^7^ CD36-KO BM cells (KO; n = 5 mice), one CD45.1 allele bearing blank mice, and one CD45.2 allele bearing blank mice. (B-C) The weights of spleens and livers were measured, and data are presented as the mean of tissue mass from WT and KO group and each colored dot represents the mass of a single mouse tissue (n = 5 mice per group). The differences between Cd36-KO and WT groups were analyzed by Mann-Whitney test (Abbreviation: ns, not significant). (D-F) Representative flow cytometry result showing BM transplant of healthy CD45.2^+^ WT or CD36-KO BM cells in the BM, spleen, and blood cells of CD45.1 allele bearing mice (n = 5 mice per group). **Figure S8.** Cd36-KO BM cells are less engrafted than WT BM cells in the competitive repopulation assay. (A-C) Representative flow cytometry result showing BM transplant and quantification of engraftment of mixed CD45.1^+^ WT and CD45.2^+^ Cd36-KO BM cells in the BM and spleen cells of CD45.1^+^ CD45.2^+^ recipient mice (n = 1 male and 1 female). The differences between groups were analyzed using ratio paired t-test (Abbreviation: *, P < 0.05). **Figure S9.** Cd36-KO mice exhibit similar AML engraftment with WT mice. (A) Spleens and livers were collected from Cd36-KO and WT mice engrafted with 5 x 10^6^ FLT3-ITD/MLL-PTD mouse leukemic cells (n = 5 mice in KO; n = 6 mice in WT), one CD45.1 allele bearing blank mice, and one CD45.2 allele bearing blank mice. (B-C) The weights of spleens and livers were measured, and data are presented as the mean of tissue mass from WT and KO group and each colored dot represents the mass of a single mouse tissue (n = 6 mice in WT group; n = 5 mice in Cd36-KO group). The differences between Cd36-KO and WT groups were analyzed by Mann-Whitney test (Abbreviation: ns, not significant). (D-G) Representative flow cytometry result showing AML engraftment of FLT3-ITD/MLL-PTD mouse leukemic cells in the BM, spleen, liver, and blood cells of WT and Cd36-KO mice (n = 6 mice in WT group; n = 5 mice in Cd36-KO group). **Figure S10. **Cd36-KO mice exhibit similar AML engraftment with WT mice. (A) Spleens and livers were collected from Cd36-KO and WT mice engrafted with 1 x 10^6^ FLT3-ITD/MLL-PTD mouse leukemic cells (n = 4 mice per group), one CD45.1 allele bearing blank mice, and one CD45.2 allele bearing blank mice. (B-C) The weights of spleens and livers were measured, and data were presented as the mean of tissue mass from WT and KO group and each colored dot represents the mass of a single mouse tissue (n = 4 mice per group). The differences of tissue weight between Cd36-KO and WT groups were analyzed by Mann-Whitney test (Abbreviation: ns, not significant). (D-G) Representative flow cytometry result showing AML engraftment and quantification of engraftment of FLT3-ITD/MLL-PTD mouse leukemic cells in the BM, spleen, liver, and blood cells of WT and Cd36-KO mice (n = 4 mice). The difference between groups were analyzed using unpaired t-test (Abbreviation: ns, not significant).

## Data Availability

All the data relevant to the study results are included in the main figures of the article or available in the supplemental information. The RNA-Seq datasets that support the findings of this study were obtained from the following databases in the public domain: Bloodspot (https://servers.binf.ku.dk/bloodspot/) and Haemosphere (https://www.haemosphere.org/).

## Abbreviations

AML: Acute myeloid leukemia
BM: Bone marrow
CBC: Complete blood count
Cd36-KO: Cd36 Knockout
CFC: Colony forming cell
CFU: Colony-forming unit
CFSE: CellTrace carboxyfluorescein succinimidyl ester
CLP: Common lymphoid progenitor
CMP: Common myeloid progenitor
GAT: Gonadal adipose tissue
GMP: Granulocyte–macrophage progenitor
Hb: Hemoglobin
HCT: Hematocrit
HSCs: Hematopoietic stem cells
HSPCs: Hematopoietic stem and progenitor cells
IL-2: Interleukin-2
LSCs: Leukemic stem cells
MCV: Mean corpuscular volume
MCH: Mean corpuscular hemoglobin
MCHC: Mean corpuscular hemoglobin concentration
MEP: Megakaryocyte-erythroid progenitor
MZ: Marginal zone
PCV: Packed cell volume
PHA: Phytohemagglutinin
RBC: Red blood cell
TIME: The tumor immune microenvironment
TPM: Transcripts per million
WBC: White blood cell
