# Supplementary material for: Deletion of CD36 exhibits limited impact on normal hematopoiesis and the leukemia microenvironment
Source: Cell Mol Biol Lett. 2023 May 24;28:45. doi: 10.1186/s11658-023-00455-8 (PMC10210361; doi:10.1186/s11658-023-00455-8)
Supplement: Supplementary file 1 — Additional file 1: Table S1. Cd36 Gene Expression in Mouse Normal Hematopoiesis. Table S2. Gene Expression for Cd36 in Mouse Normal Hematopoiesis. Table S3. Gene Expression for CD36 in Human Normal Hematopoiesis. Table S4. Cd36 expression in mouse normal hematopoietic system. Table S5. CD36 expression in normal human Hematopoiesis. Figure S1. Cd36 differential gene expression patterns in normal hematopoiesis between mouse and human. (A-B) Log2 transformed gene expression level for Cd36 in mouse normal hematopoietic system obtained from GSE14833 and GSE6506 datasets and CD36 expression in normal human hematopoiesis obtained from datasets GSE17054, GSE19599, GSE11864, and E-MEXP-1242, which all datasets were downloaded from BloodSpot database. Data are presented as the mean of gene expression among each cell population and each colored dot represents the expression value of a single sample. Unpaired t-test analysis was used (***, P < 0.001; **, P < 0.01; *, P < 0.05). Figure S2. Confirmation of Cd36 reduced expression in Cd36-KO mice compared with WT mice. (A) Genotype confirmation of wildtype and Cd36 knockout mice by gel electrophoresis of DNA fragments generated by standard PCR with recommended primers. Lane 1 and 2: WT mice liver tissues with WT primers; Lane 3 and 4: WT liver mice tissues with Cd36-KO primers; Lane 5 and 6: Cd36-KO liver mice tissues with WT primers; Lane 7 and 8: Cd36-KO mice tissues with Cd36-KO primers. (B-D) qPCR quantification of Cd36 knockout efficiency in healthy mouse bone marrow cells, spleen cells, and liver cells (n = 6 mice per group). The bar graph represents the mean of Cd36 mRNA level in per group with standard error of the mean. Welch's t-test was used to analyze the significant difference (**, P < 0.01). (E-H) Cd36 knockout was confirmed using flow cytometry by comparing the cell surface Cd36+ population in bone marrow, spleen, liver, and blood tissues (n = 6 mice per group). (I) The spleens and livers were collected from Cd36-KO [file 11658_2023_455_MOESM1_ESM.pdf]

Additional file 1:

TABLE S1. *CD36* GENE EXPRESSION IN MOUSE NORMAL HEMATOPOIESIS

**Abbreviation**	**Full Name**	**Log2 Expression**					
LT-HSC	Long Term Hematopoietic Stem Cell	3.18					
HSC	Hematopoietic Stem Cell	0.79	0.79	0.79	0.79		
MPP	Multipotent Progenitor	0.79	0.79				
CLP	Common Lymphoid Progenitor	1.53	0.79	1.54	1.50		
CMP	Common Myeloid Progenitor	2.05	1.90	2.04	0.79		
GMP	Granulocyte Monocyte Progenitor	0.79	1.52	0.79	0.79		
Macro	Bone Marrow Macrophages	2.33	1.62	6.83	6.38		
Granu	Granulocytes	2.99	3.04	0.79	4.37	4.33	3.94
Mono	Monocytes	4.86	4.17	3.59	3.46		
B Cells	B cells	5.91	5.76				
T_CD4+	T CD4+ cells	0.79	0.79	0.79	0.79		
T_CD8+	T CD8+ cells	2.05	2.21	0.79	0.79		
NK	NK Cells	6.61	6.88				
MEP	Megakaryocitic erythroyd progenitor	2.99	3.06	2.51	2.78		
Ery_A	Erythrocytes A	7.33	7.12				
Ery_B	Erythrocytes B	8.46					

TABLE S2. GENE EXPRESSION FOR *CD36* IN MOUSE NORMAL HEMATOPOIESIS

Cell Lineage	Cell Type	Expression Value					
Multi Potential Progenitor	STHSC	0.21	0.23				
	LSK	0.62	0.76				
	MPP	0.35	0.38				
Restricted Potential Progenitor	CMP	0.60	0.31				
	PreGMFlt3Neg	0.42	0.90				
	PreGMFlt3Pos	0.36	0.44				
	GMP	0.17	0.29				
	GMP_IRF8lo	1.77	2.07				
	GMP_IRF8int	1.64	1.25				
	GMP_IRF8hi	0.28	0.50				
	GMPSigF	0.11	0.83	0.94	0.67	0.84	
	FcgRCD150	1.67	1.89				
	CD9Hi	0.56	0.67				
	BEMP	0.85	0.65				
	CLP	0.85	1.52				
Erythrocyte Lineage	PreCFUE	3.42	3.31				
	MEP	3.52	3.46				
	CFUE	3.59	3.83				
	EryBlPB	8.26	8.06	9.44			
	EryBlPO	10.18	10.58	7.93			
	Retic	6.12	6.39	6.72			
Megakaryocyte Lineage	MegTPO	0.03	0.24				
Mast Cell Lineage	Mast	3.36	4.89				
Basophil Lineage	BasoBM	0.27	0.00				
	BasoSpl	0.51					
	BasoCult	4.04	3.42				
Eosinophil Lineage	EoP	0.24	0.69				
	Eo	0.69	0.93	0.67			
	EoSSCLo	0.27	0.06	0.06	0.11	0.30	
	EoSSCHi	0.09	0.00	0.08	0.23	0.12	0.20
	EoCult	0.49	0.88				
Neutrophil Lineage	NeutBM	0.08	0.00				
	NeutPB	1.43	0.77	1.12	1.64		
Macrophage Lineage	MonoBM	2.03	1.28				
	MonoPB	3.99	3.83				
	Mac	9.40	9.46				
	InfMono	1.36	1.32				
	MacCult	8.42	8.67	8.67			
Dendritic Cell Lineage	cDC2	3.53	3.70				
	pDC	2.83	2.81				
B Cell Lineage	Fob	5.72	4.92				
	MZB	7.66	7.43				
	B1Pt	5.13	5.87				
	PtB	5.77	5.69				
	GCB	4.32	4.86				
	SplPlsB	3.96					
	SplPlsC	1.40	1.79	1.68			
	BMPlsC	0.48					
	NveCD4T	0.06	0.00				

	NveCd8T	0.06	0.06
T Cell Lineage	CD4T	0.00	0.00
	CD8T	1.00	0.05
	EffCD4T	0.07	0.13
	RegT	0.37	0.58
	MemCD4T	0.36	0.00
	MemCD8T	0.53	0.17
NK Cell Lineage	NK	0.00	0.30

TABLE S3. GENE EXPRESSION FOR *CD36* IN HUMAN NORMAL HEMATOPOIESIS

Cell Lineage	Cell Type	Expression Value				
B cell lineage	Memory B cell	0.18	0.28	0.13		
	Naïve B Cell	0.13	0.17	0.18	0.05	0.17
Dendritic Cell Lineage	Myeloid Dendritic Cell	0.28	0.09	3.62		
	CD123+ Myeloid Dendritic Cell	3.00	4.45			
	Plasmacytoid Dendritic Cell	7.62	7.37			
Eosinophil Lineage	Eosinophil	0.09	0.82			
Macrophage Lineage	Monocyte	5.92	6.56	6.47	5.46	6.51
	Non-classical monocyte	3.73	3.11			
Neutrophil Lineage	Neutrophil	0.62	0.18	0.24		
NK Cell Lineage	Natural Killer Cell	1.93	2.98	3.00	1.86	4.58
T Cell Linage	CD4+ T Cell	0.26	0.13	0.18	0.09	0.08
	CD8+ T Cell	0.16	0.12	0.18	0.04	0.23

TABLE S4. *CD36* EXPRESSION IN MOUSE NORMAL HEMATOPOIETIC SYSTEM

Cell lineage	Cell type	Log2 expression				
Multi potential progenitor	Long term hematopoietic stem cell	3.54	3.83	3.61	3.92	
	Short term hematopoietic stem cell	3.25	3.50			
	Lymphoid-primed multipotential progenitors	3.37	3.17	3.62	3.76	3.99
Restricted potential progenitor	Common lymphoid progenitor cells	3.28	3.32			
	Granulocyte monocyte progenitors	3.22	3.30	3.25		
Erythrocyte lineage	Erythroid progenitor cells	7.44	6.83	6.79		
	Nucleated erythrocytes	9.74	10.6 1			
	Pre-colony-forming unit erythroid cells	5.02	4.17	3.41		
	Colony-forming unit erythroid cells	5.51	5.84	4.28		
Megakaryocyte lineage	Megakaryocyte precursor	3.12	3.43	3.39		
	Megakaryocyte erythroid precursors	3.38	3.25	3.40	3.43	
Granulocyte-monocyte lineage	Pre-granulocyte monocyte	3.14	3.36			
	Granulocytes	4.93	3.91			
	Monocyte	5.61	7.61			
B cell lineage	Pre-b cell	5.39	5.59	5.58	4.57	
	Pro-b cell	3.61	3.68			
	B-cell	6.47	6.77			
	Immunoglobulin m positive side population cells	6.11	7.04			
T cell lineage	Early t-cell progenitor	3.54	3.37	3.10		
	Cd4 t-cells	3.88	3.69	3.53	3.93	
	Activated cd4 positive t-cells	3.64	3.82			
	Naive cd8 positive t-cells	4.19	4.59			
	Activated cd8 positive t-cells	3.57	3.39			
Nk cell lineage	Cd56+ natural killer cells	3.51	3.32			
	Mature natural killer cells	3.28	2.98			

TABLE S5. *CD36* EXPRESSION IN NORMAL HUMAN HEMATOPOIESIS

Cell lineage	Cell Type	Log2 Expression				
**Multi potential progenitor**	Hematopoietic progenitor cells	7.21	9.03	6.40	4.73	
	Hematopoietic stem cells	3.88	3.26	3.42	4.18	
		3.51	3.35	4.69	3.13	
**Restricted potential progenitor**	Common myeloid progenitor cell	5.53	5.29	9.94		
	Granulocyte monocyte progenitors	5.82	5.94	5.95		
	Megakaryocyte-erythroid progenitor cell	9.83	10.85	8.73		
**Myocyte lineage**	Promyelocyte	4.88	4.96	6.80		
	Myelocyte	4.80	2.96	3.08		
	Polymorphonuclear cells from bone marrow	3.61	3.17	3.09		
	Polymorphonuclear cells from peripheral blood	3.28	3.35	3.29		
**Monocyte lineage**	Monocytes	11.96	10.70	10.40	10.80	10.59
		10.67	10.71	10.60	10.87	11.36
		11.19	11.36	11.32	11.25	
**Dendritic cell lineage**	Myeloid dendritic cells	8.68	9.00	8.81	8.48	8.51
	Plasmacytoid dendritic cells	9.93	10.25	9.93	9.93	10.03
**B cell lineage**	B cells	3.16	3.82	3.97	3.65	3.81
**T cell lineage**	CD4+ T cells	6.12	9.72	8.31	6.10	6.40
	CD8+ T cells	3.62	3.55	3.49	3.56	3.58
**Nk cell lineage**	NK cells	3.74	3.98	4.07	3.55	3.50

Figure S1. *Cd36* differential gene expression patterns in normal hematopoiesis between mouse and human

A

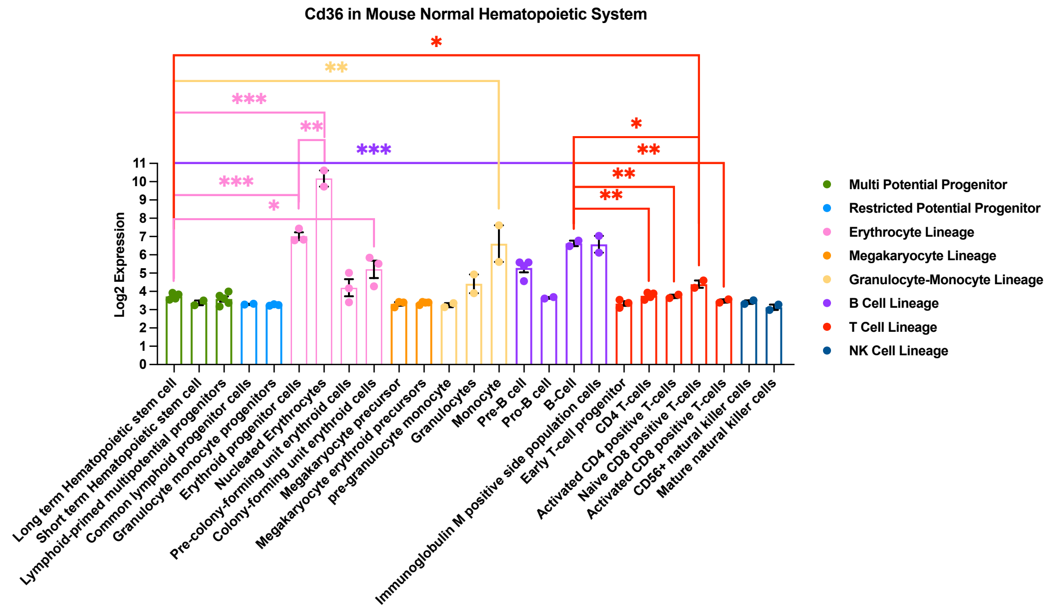

B

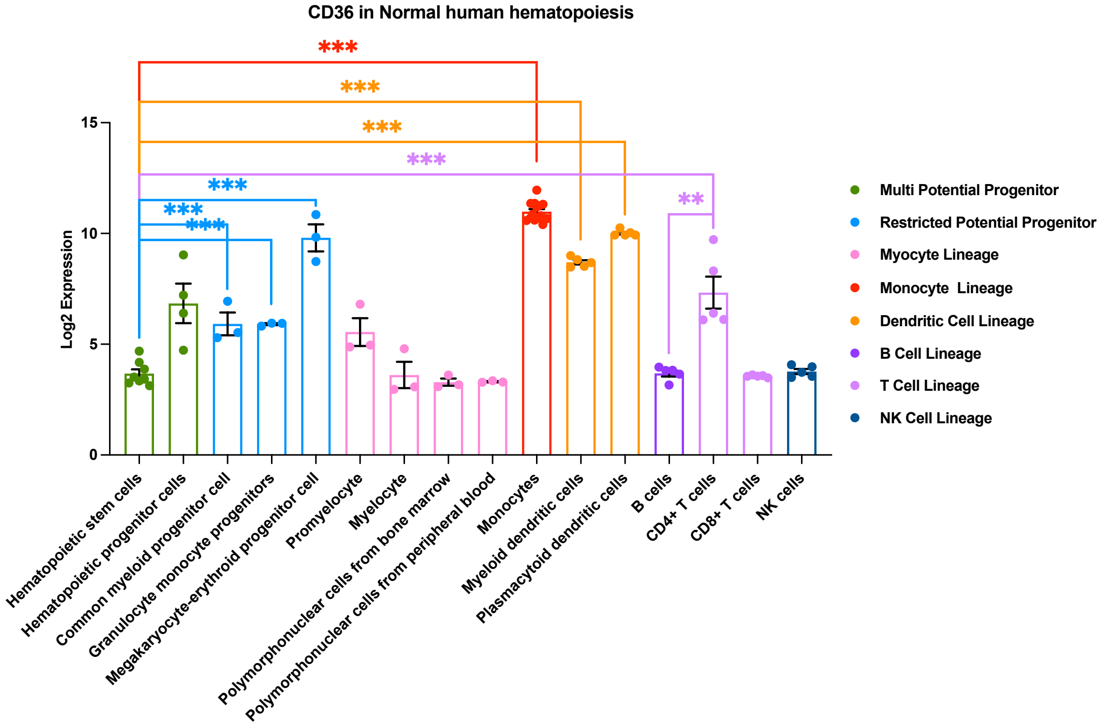

Figure S1. *Cd36* differential gene expression patterns in normal hematopoiesis between mouse and human. (A-B) Log2 transformed gene expression level for *Cd36* in mouse normal hematopoietic system obtained from GSE14833 and GSE6506 datasets and *CD36* expression in normal human hematopoiesis obtained from datasets GSE17054, GSE19599, GSE11864, and E-MEXP-1242, which all datasets were downloaded from BloodSpot database. Data is presented as the mean of gene expression among each cell population and each colored dot represents the expression value of a single sample. Unpaired t-test analysis was used (***, $P < 0.001$; **, $P < 0.01$; *, $P < 0.05$).

Figure S2. Confirmation of *Cd36* reduced expression in Cd36-KO mice compared with WT mice

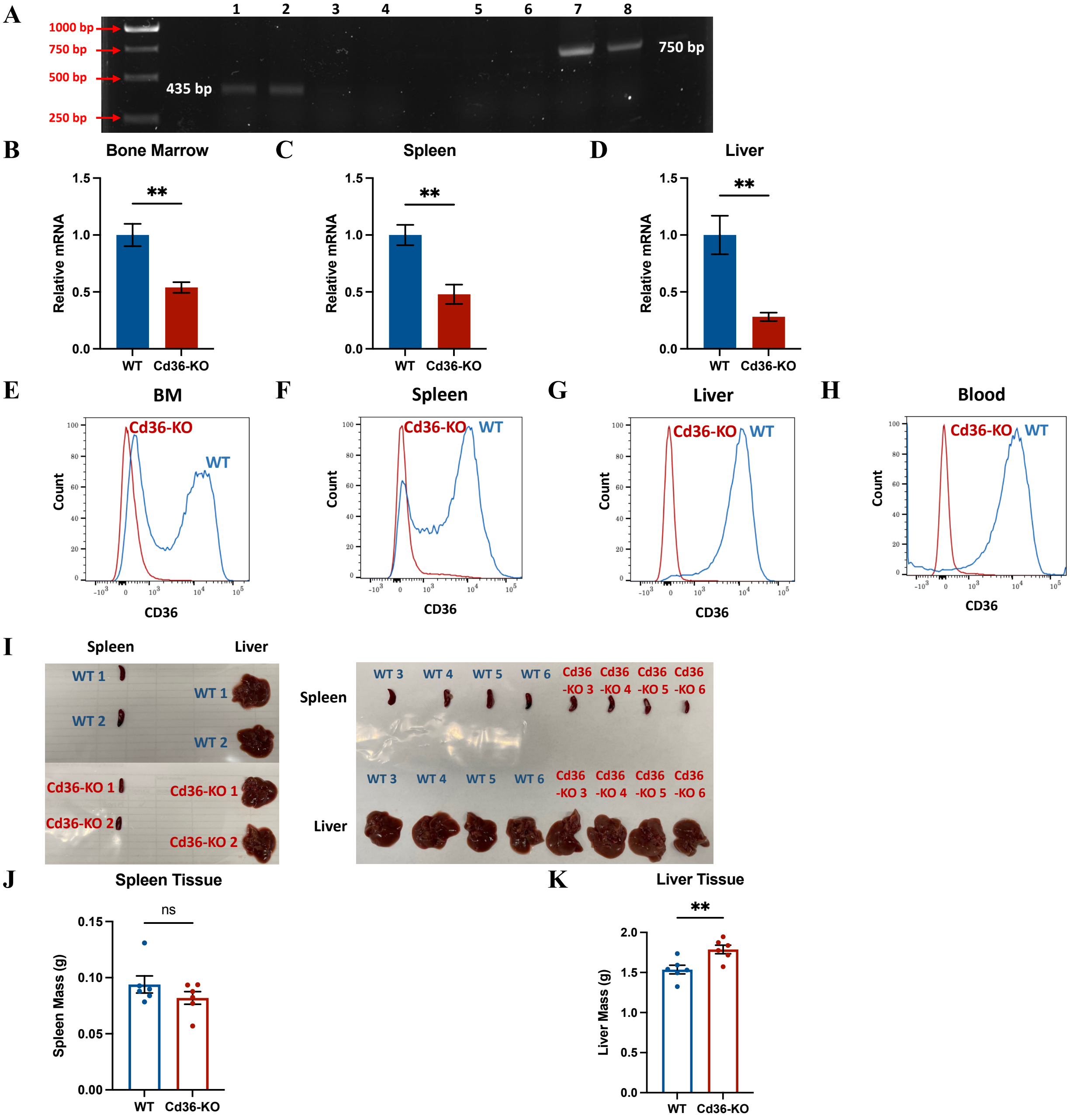

L

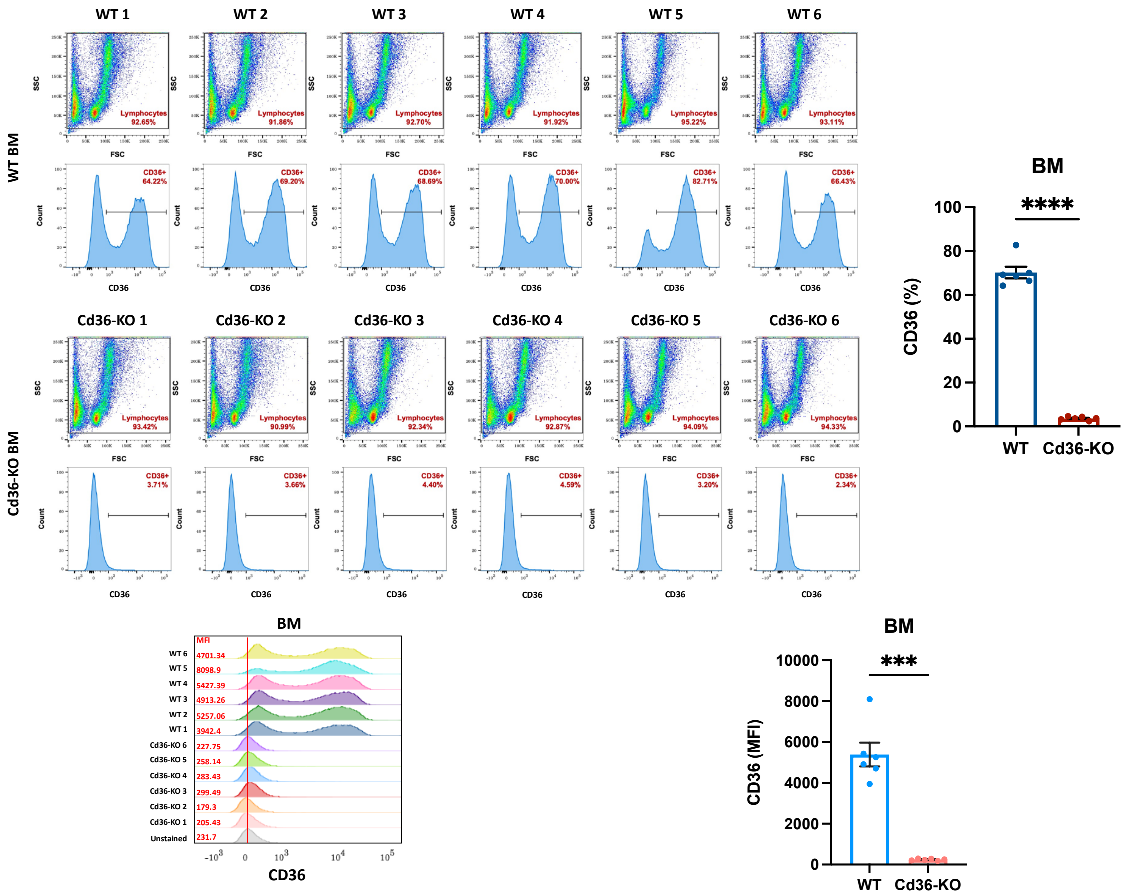
WT 1
WT 2
WT 3
WT 4
WT 5
WT 6
WT BM
Lymphocytes 92.65%
Lymphocytes 91.86%
Lymphocytes 92.70%
Lymphocytes 91.92%
Lymphocytes 95.22%
Lymphocytes 93.11%
CD36+ 64.22%
CD36+ 69.20%
CD36+ 68.69%
CD36+ 70.00%
CD36+ 82.71%
CD36+ 66.43%
Cd36-KO 1
Cd36-KO 2
Cd36-KO 3
Cd36-KO 4
Cd36-KO 5
Cd36-KO 6
Cd36-KO BM
Lymphocytes 93.42%
Lymphocytes 90.99%
Lymphocytes 92.34%
Lymphocytes 92.87%
Lymphocytes 94.09%
Lymphocytes 94.33%
CD36+ 3.71%
CD36+ 3.66%
CD36+ 4.40%
CD36+ 4.59%
CD36+ 3.20%
CD36+ 2.34%
SSC
FSC
Count
CD36
BM
****
CD36 (%)
WT
Cd36-KO
BM
MFI
WT 6 4701.34
WT 5 8098.9
WT 4 5427.39
WT 3 4913.26
WT 2 5257.06
WT 1 3942.4
Cd36-KO 6 227.75
Cd36-KO 5 258.14
Cd36-KO 4 283.43
Cd36-KO 3 299.49
Cd36-KO 2 179.3
Cd36-KO 1 205.43
Unstained 231.7
CD36
BM
***
CD36 (MFI)
WT
Cd36-KO

M

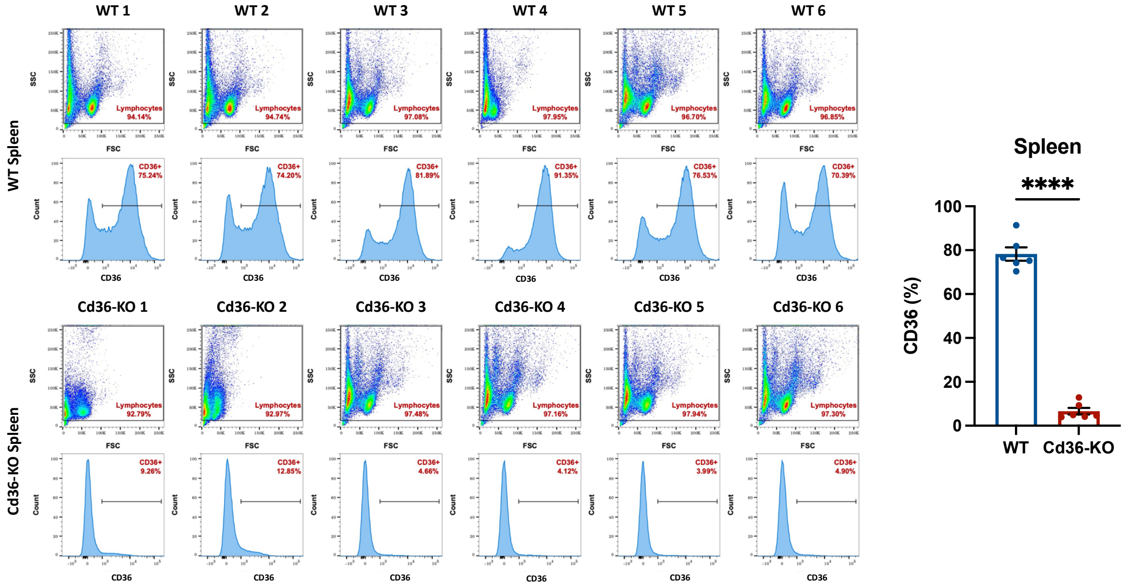
WT 1
WT 2
WT 3
WT 4
WT 5
WT 6
WT Spleen
Lymphocytes 94.14%
Lymphocytes 94.74%
Lymphocytes 97.08%
Lymphocytes 97.95%
Lymphocytes 96.70%
Lymphocytes 96.85%
CD36+ 75.24%
CD36+ 74.20%
CD36+ 81.89%
CD36+ 91.35%
CD36+ 76.53%
CD36+ 70.39%
Cd36-KO 1
Cd36-KO 2
Cd36-KO 3
Cd36-KO 4
Cd36-KO 5
Cd36-KO 6
Cd36-KO Spleen
Lymphocytes 92.79%
Lymphocytes 92.97%
Lymphocytes 97.48%
Lymphocytes 97.16%
Lymphocytes 97.94%
Lymphocytes 97.30%
CD36+ 9.26%
CD36+ 12.85%
CD36+ 4.66%
CD36+ 4.12%
CD36+ 3.99%
CD36+ 4.90%
SSC
FSC
Count
CD36
Spleen
****
CD36 (%)
WT
Cd36-KO

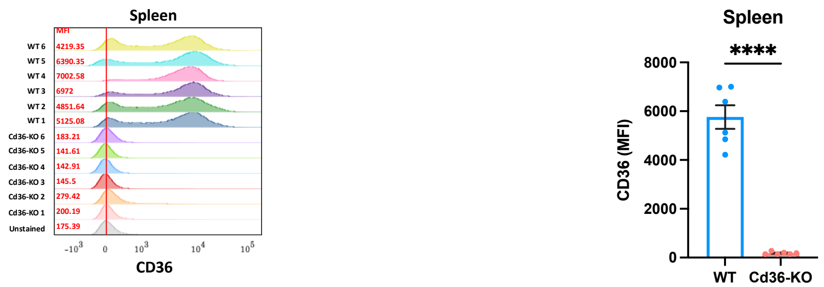
Spleen
MFI
WT 6 4219.35
WT 5 6390.35
WT 4 7002.58
WT 3 6972
WT 2 4851.64
WT 1 5125.08
Cd36-KO 6 183.21
Cd36-KO 5 141.61
Cd36-KO 4 142.91
Cd36-KO 3 145.5
Cd36-KO 2 279.42
Cd36-KO 1 200.19
Unstained 175.39
CD36
Spleen
****
CD36 (MFI)
8000
6000
4000
2000
0
WT
Cd36-KO

N

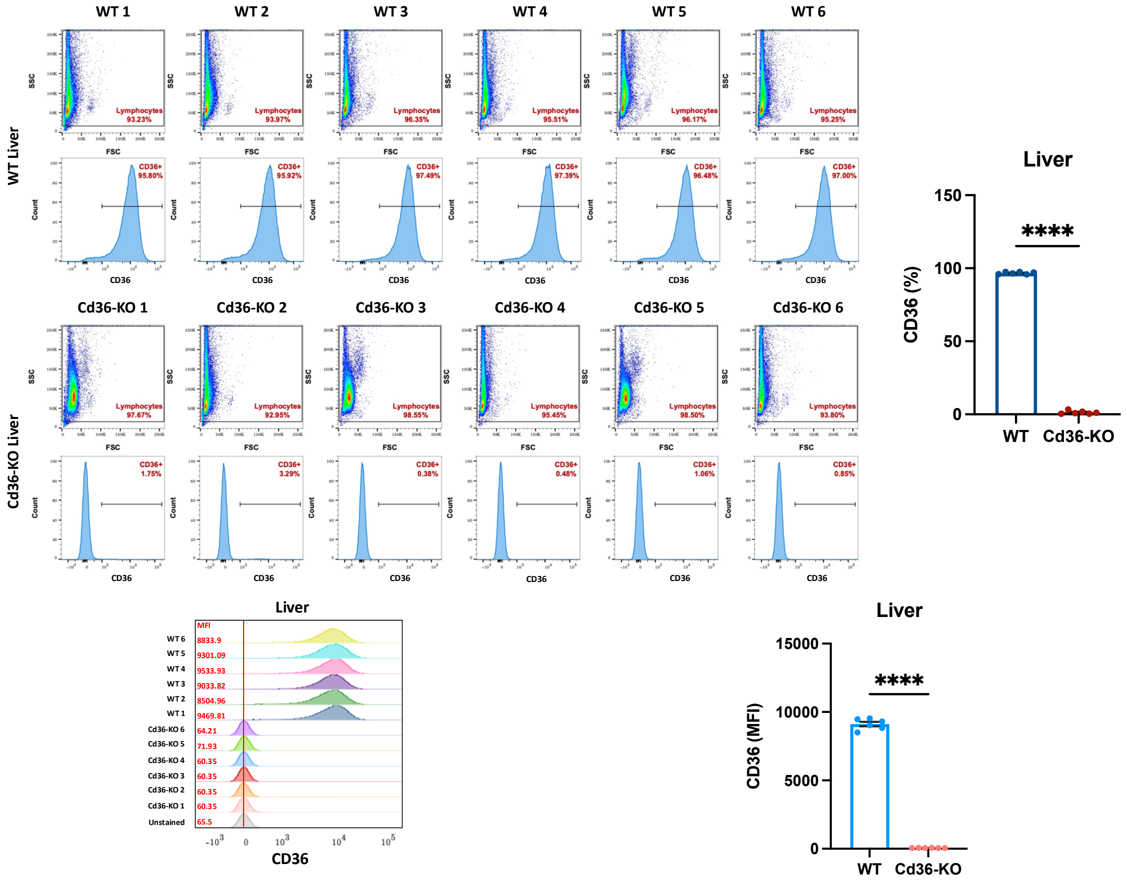
WT 1
WT 2
WT 3
WT 4
WT 5
WT 6
WT Liver
SSC
FSC
Lymphocytes 93.23%
Lymphocytes 93.97%
Lymphocytes 96.35%
Lymphocytes 95.51%
Lymphocytes 96.17%
Lymphocytes 95.25%
Count
CD36
CD36+ 95.80%
CD36+ 95.92%
CD36+ 97.49%
CD36+ 97.39%
CD36+ 96.48%
CD36+ 97.00%
Cd36-KO 1
Cd36-KO 2
Cd36-KO 3
Cd36-KO 4
Cd36-KO 5
Cd36-KO 6
Cd36-KO Liver
Lymphocytes 97.67%
Lymphocytes 92.95%
Lymphocytes 98.55%
Lymphocytes 95.45%
Lymphocytes 98.50%
Lymphocytes 93.80%
CD36+ 1.75%
CD36+ 3.29%
CD36+ 0.38%
CD36+ 0.48%
CD36+ 1.06%
CD36+ 0.85%
Liver
****
CD36 (%)
150
100
50
0
WT
Cd36-KO
Liver
MFI
WT 6 8833.9
WT 5 9301.09
WT 4 9533.93
WT 3 9033.82
WT 2 8504.96
WT 1 9469.81
Cd36-KO 6 64.21
Cd36-KO 5 71.93
Cd36-KO 4 60.35
Cd36-KO 3 60.35
Cd36-KO 2 60.35
Cd36-KO 1 60.35
Unstained 65.5
CD36
Liver
****
CD36 (MFI)
15000
10000
5000
0
WT
Cd36-KO

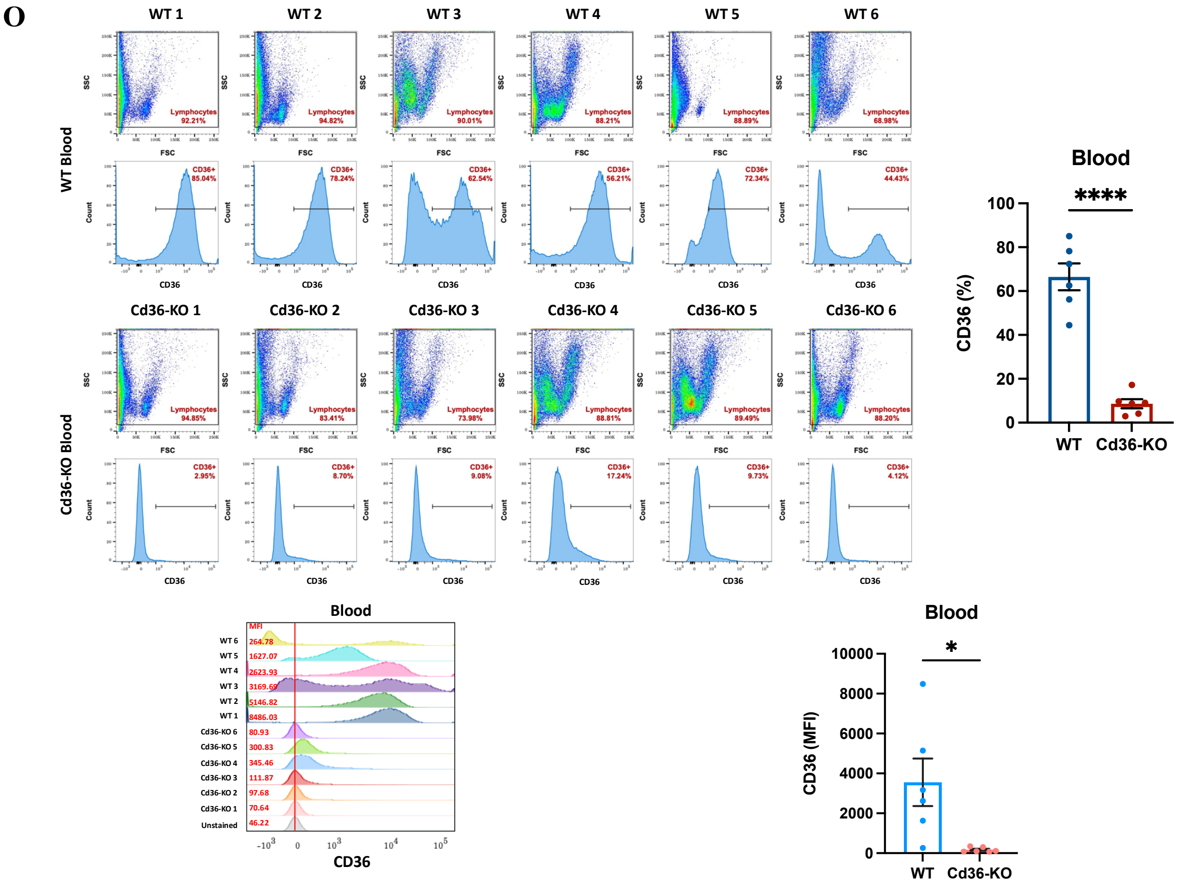

Figure S2. Confirmation of *Cd36* reduced expression in Cd36-KO mice compared with WT mice

(A) Genotype confirmation of wildtype and *Cd36* knockout mice by gel electrophoresis of DNA fragments generated by standard PCR with recommended primers. Lane 1 and 2: WT mice liver tissues with WT primers; Lane 3 and 4: WT liver mice tissues with Cd36-KO primers; Lane 5 and 6: Cd36-KO liver mice tissues with WT primers; Lane 7 and 8: Cd36-KO mice tissues with Cd36-KO primers.

(B-D) qPCR quantification of *Cd36* knockout efficiency in healthy mouse bone marrow cells, spleen cells, and liver cells (n = 6 mice per group). The bar graph represents the mean of *Cd36* mRNA level in per group with standard error of the mean. Welch's t-test was used to analyze the significant difference (**, P < 0.01).

(E-H) *Cd36* knockout was confirmed using flow cytometry by comparing the cell surface Cd36$^+$ population in bone marrow, spleen, liver, and blood tissues (n = 6 mice per group).

(I) The spleens and livers were collected from Cd36-KO and WT mice.

(J-K) The weight of spleen and liver organs were compared. The bar graph represents the mean of spleen and liver mass in Cd36-KO and WT mice with standard error of the mean. Each colored dot represents the mass value of every single mouse tissue (n = 6 mice per group). The differences between Cd36-KO and WT mice tissues were analyzed by unpaired t-test (**, P < 0.01; Abbreviation: ns, not significant).

(L-O) *Cd36* knockout efficiency was confirmed using flow cytometry analysis by comparing the cell surface Cd36$^+$ population and *Cd36* MFI in BM, spleen, liver, and blood cells. Data is presented as the mean of Cd36$^+$ surface expression or *CD36* MFI and each colored dot represents the value of a single sample. (n = 6 mice per group). The differences between Cd36-KO and WT mice tissues were analyzed by Welch's t-test (*, P < 0.05; ***, P < 0.001; ****, P < 0.0001).

Figure S3. Hematological analysis reveals similar blood counts between Cd36-KO and WT mice

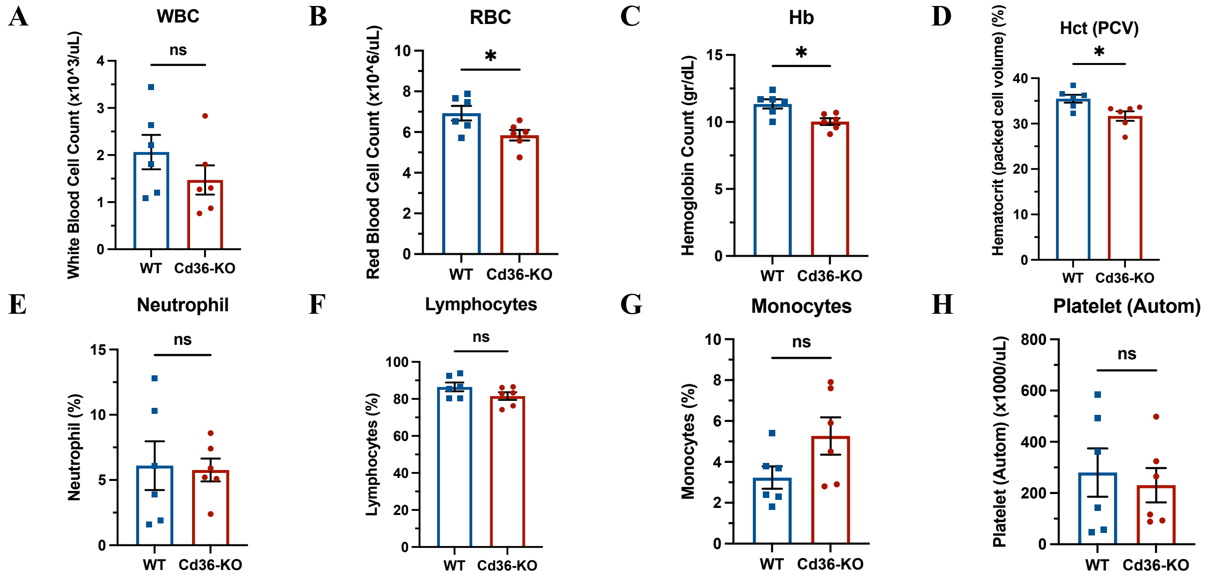

Figure S3. Hematological analysis reveals similar blood counts between Cd36-KO and WT mice (A-H) Data is presented as the mean of blood count between Cd36-KO mice and WT mice for white blood cell, red blood cell, hemoglobin, hematocrit, neutrophil, lymphocyte, monocyte, and platelet. Each colored single dot represents the count for every single mouse (n = 6 mice per group). The differences between Cd36-KO and WT group were analyzed by unpaired t-test (*, $P < 0.05$; Abbreviation: ns, not significant).

Figure S4. Cd36-KO mice exhibit similar T cell phenotypes compared with WT mice

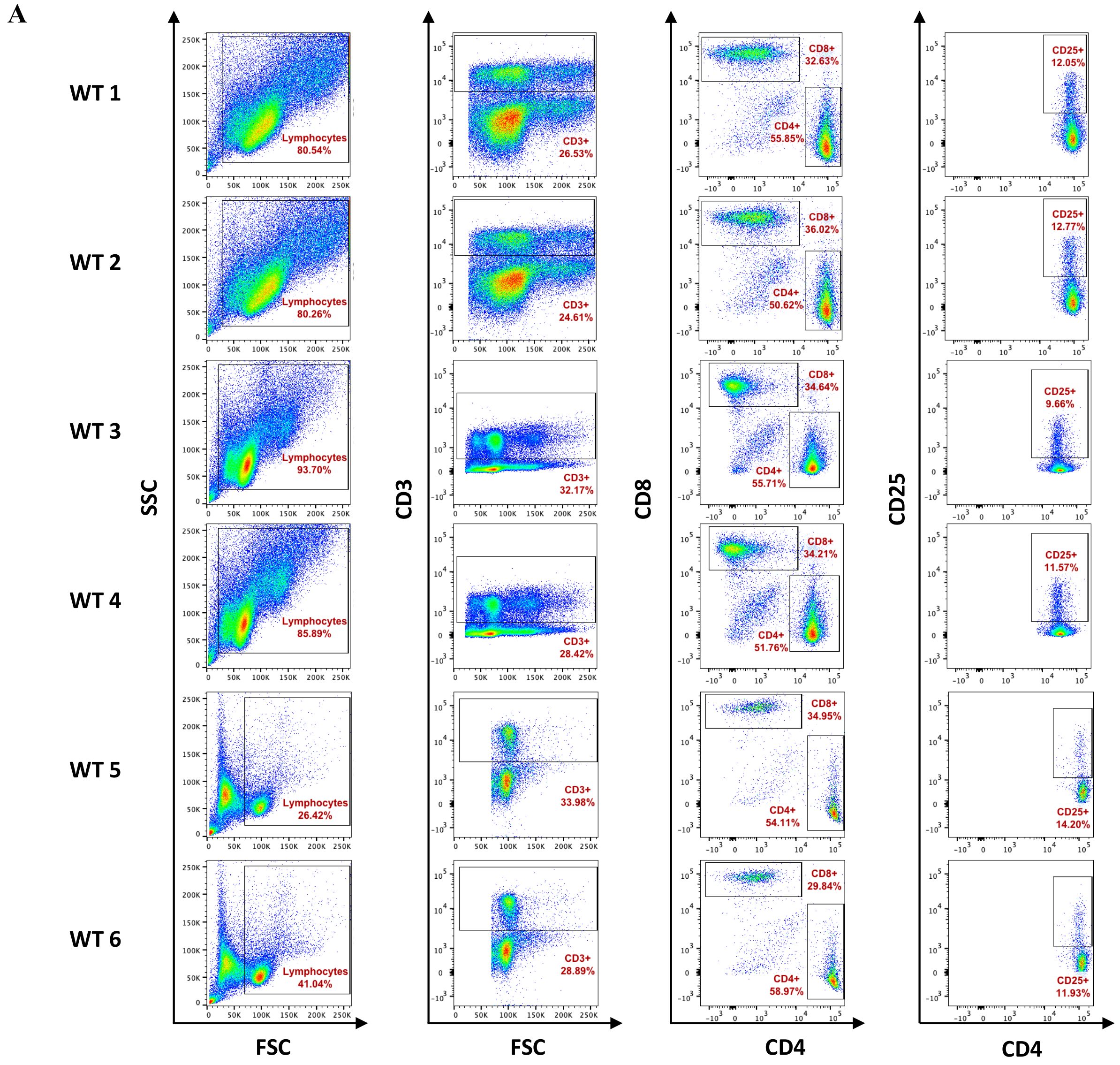

B

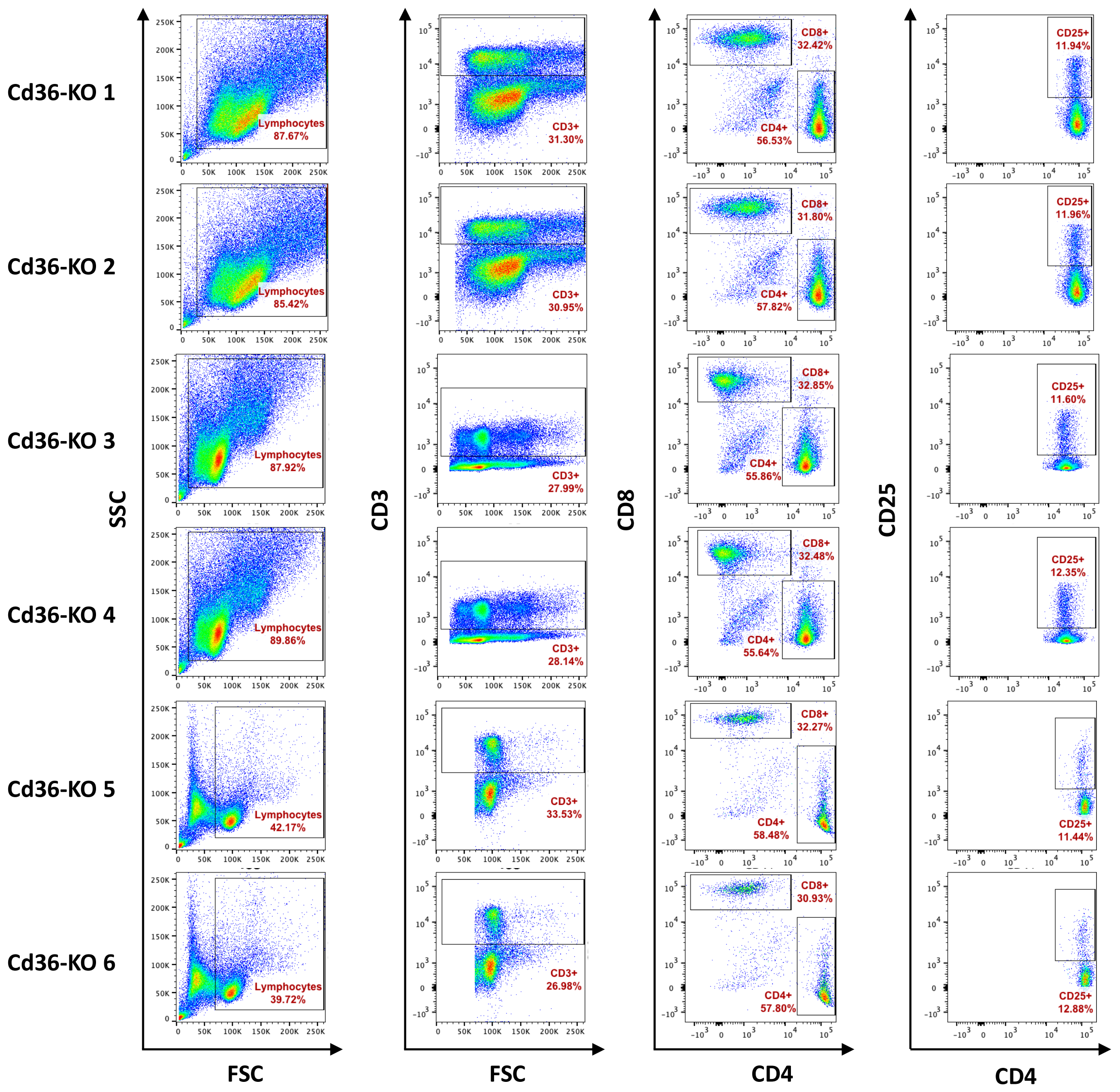

Figure S4. Cd36-KO mice exhibit similar T cell phenotypes compared with WT mice

(A-B) Representative flow cytometry of the lymphocytes population from mouse spleen cells stained with $CD3^+$, $CD4^+$, $CD8^+$, and $CD25^+$ flow antibody to evaluate the different T cell population in WT mice and Cd36-KO mice (n = 6 mice per group).

Figure S5. Chracterization of hematopoietic stem and progenitors in WT and Cd36-KO mice

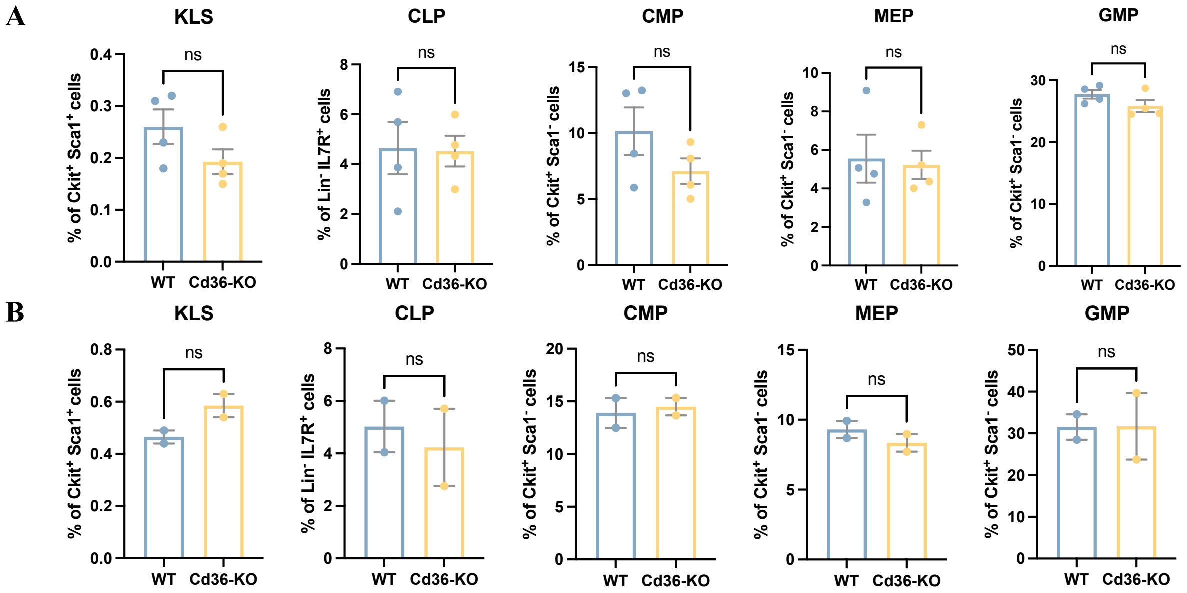

Figure S5. Chracterization of hematopoietic stem and progenitors in WT and Cd36-KO mice

(A) Quantification results of the positive cell population percentages in freshly unriched BM cells were represented by the bar graph, in which each bar represents the mean with standard error of population percentage for Cd36-KO and WT mice (n = 4 female mice per group). Shown here are an early form of murine hematopoietic stem cell (KLS), common lymphoid progenitor (CLP), common myeloid progenitor (CMP), megakaryocyte-erythroid progenitor (MEP), and granulocyte-macrophage progenitor (GMP). The differences between groups were analyzed using unpaired t-test (Abbreviation: ns, not significant).

(B) Quantification results of the positive cell population percentages in frozen unriched BM cells were represented by the bar graph, in which each bar represents the mean with standard error of population percentage for Cd36-KO and WT mice (n = 2 male mice per group). Shown here are an early form of murine hematopoietic stem cell (KLS), common lymphoid progenitor (CLP), common myeloid progenitor (CMP), megakaryocyte-erythroid progenitor (MEP), and granulocyte-macrophage progenitor (GMP). The differences between groups were analyzed using unpaired t-test (Abbreviation: ns, not significant).

Figure S6. The homing rate was similar between WT and Cd36-KO mice

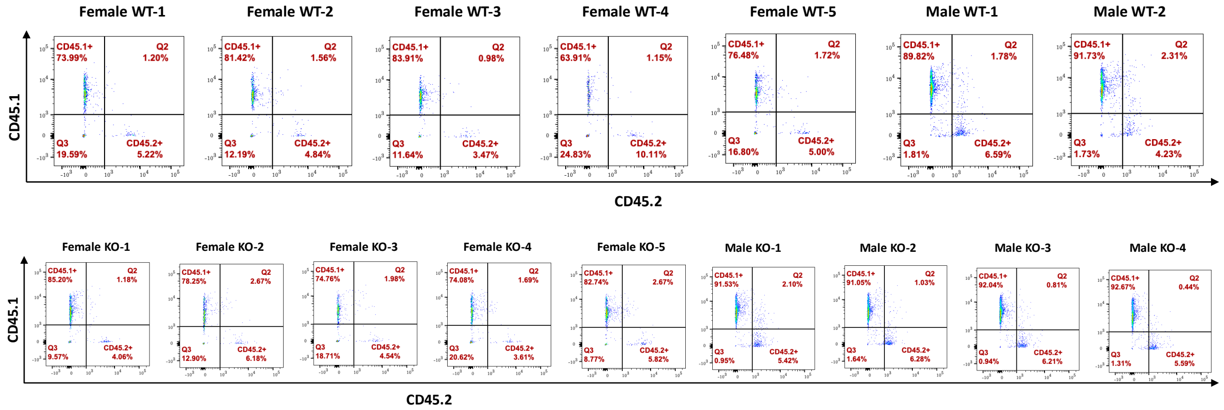

Figure S6. The homing rate was similar between WT and Cd36-KO mice

Representative flow cytometry result showing CD45.2$^+$ WT or Cd36-KO HSPCs homing in CD45.1$^+$ recipient BM niches (n = 7 in WT including 5 female and 2 male in WT group and n = 9 in Cd36-KO including 5 female and 4 male).

Figure S7. ***Cd36*** **is dispensable for normal HSPCs homing and engraftment in the bone marrow**

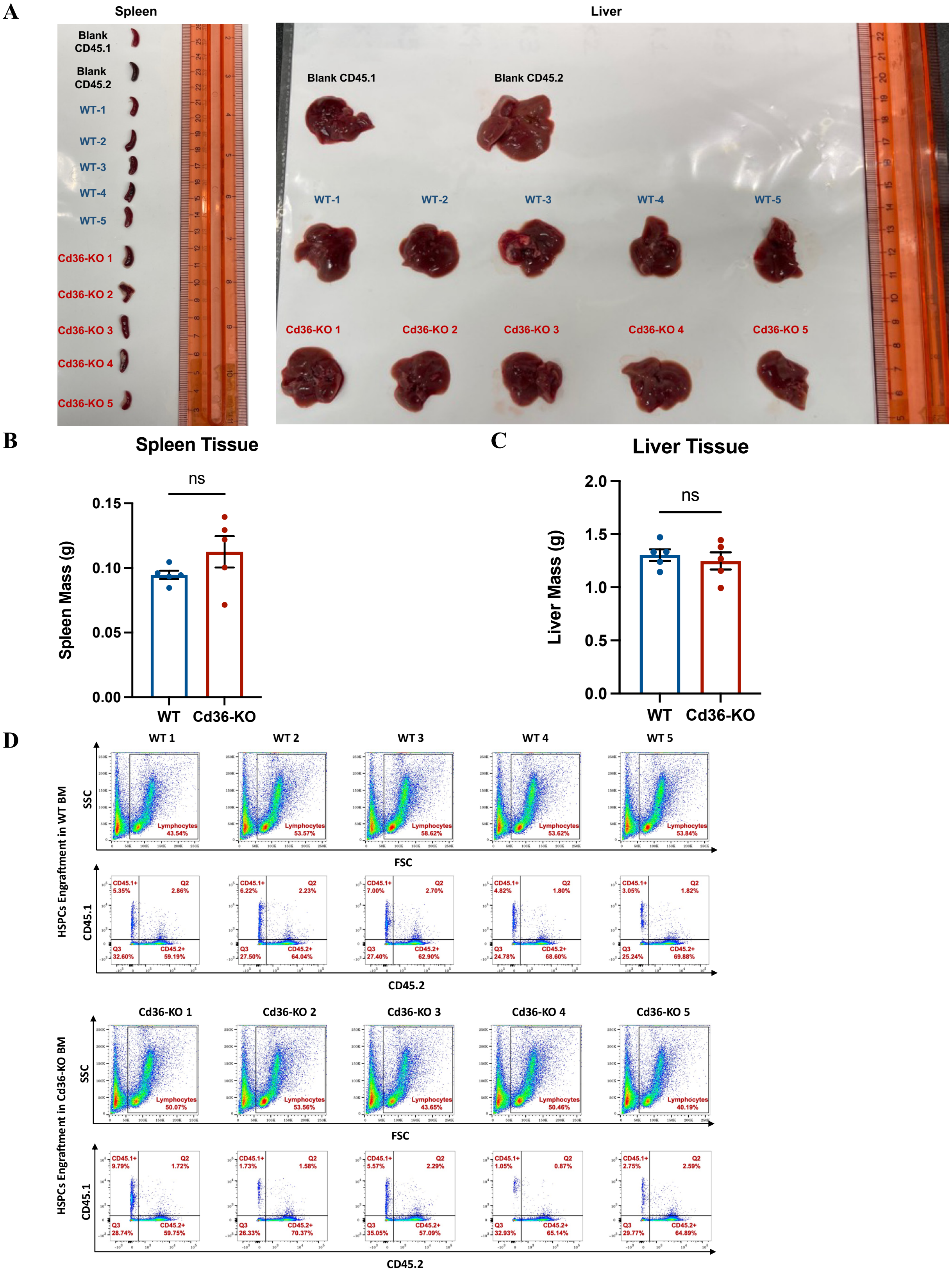

E

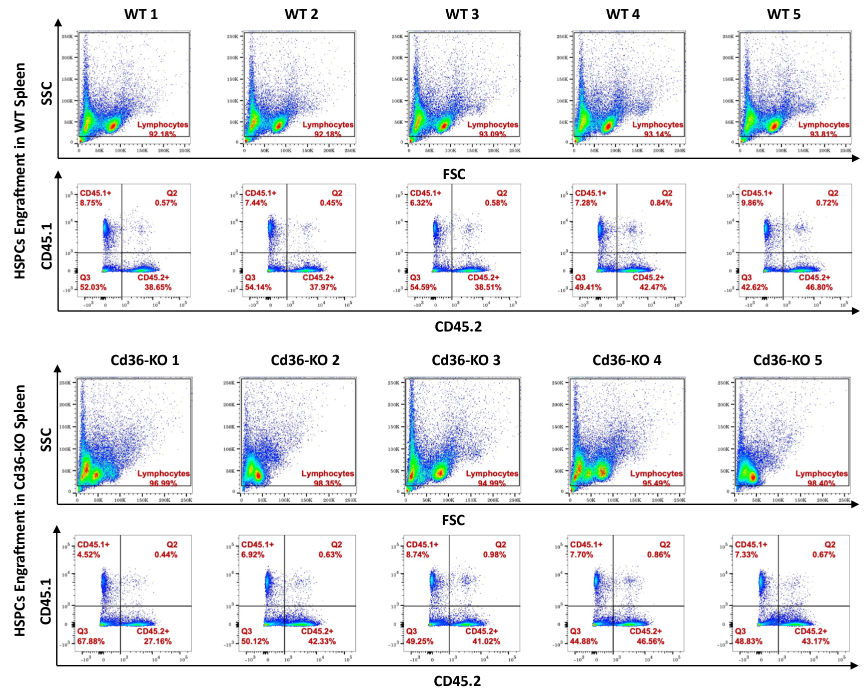

F

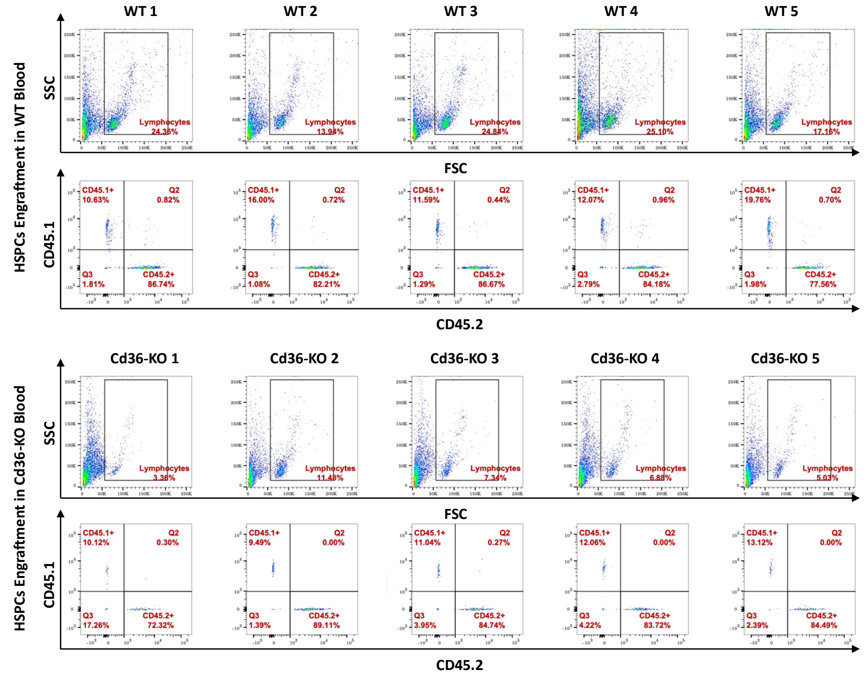

Figure S7. Cd36 is dispensable for normal HSPCs homing and engraftment in the bone marrow

(A) Spleens and livers were collected from CD45.1 allele bearing mice injected with either 2.4 x 10^7 $CD45.2^+$ BM cells (WT; n = 5 mice) or 2.4 x 10^7 CD36-KO BM cells (KO; n = 5 mice), one CD45.1 allele bearing blank mice, and one CD45.2 allele bearing blank mice.

(B-C) The weights of spleens and livers were measured, and data is presented as the mean of tissue mass from WT and KO group and each colored dot represents the mass of a single mouse tissue (n = 5 mice per group). The differences between Cd36-KO and WT groups were analyzed by Mann-Whitney test (Abbreviation: ns, not significant).

(D-F) Representative flow cytometry result showing BM transplant of healthy $CD45.2^+$ WT or CD36-KO BM cells in the BM, spleen, and blood cells of CD45.1 allele bearing mice (n = 5 mice per group).

Figure S8. Cd36-KO BM cells are less engrafted than WT BM cells in the competitive repopulation assay

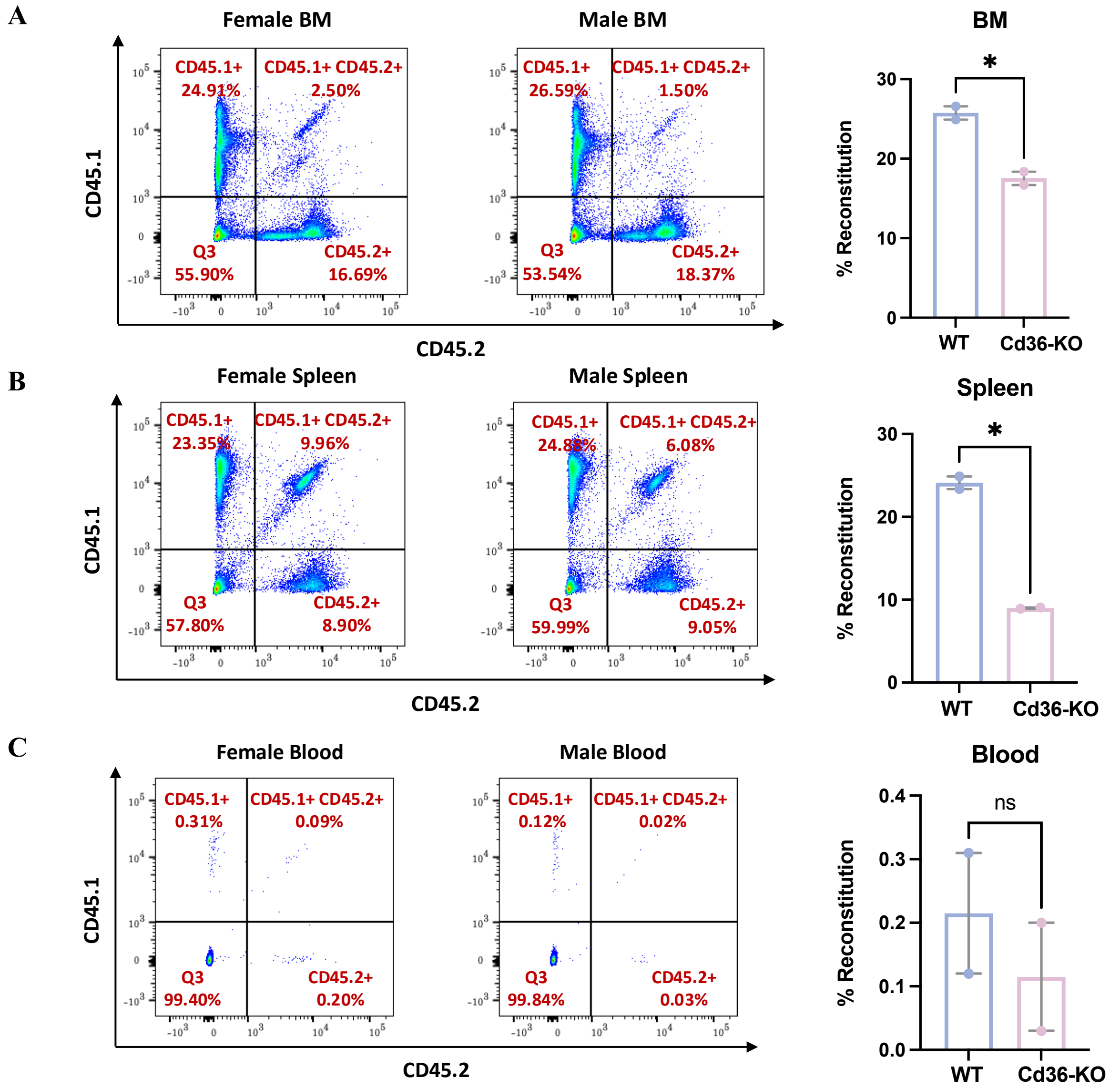

Figure S8. Cd36-KO BM cells are less engrafted than WT BM cells in the competitive repopulation assay

(A-C) Representative flow cytometry result showing BM transplant and quantification of engraftment of mixed CD45.1^{+} WT and CD45.2^{+} Cd36-KO BM cells in the BM and spleen cells of CD45.1^{+} CD45.2^{+} recipient mice (n = 1 male and 1 female). The differences between groups were analyzed using ratio paired t-test (Abbreviation: *, P < 0.05)

Figure S9. Cd36-KO mice exhibit similar AML engraftment with WT mice

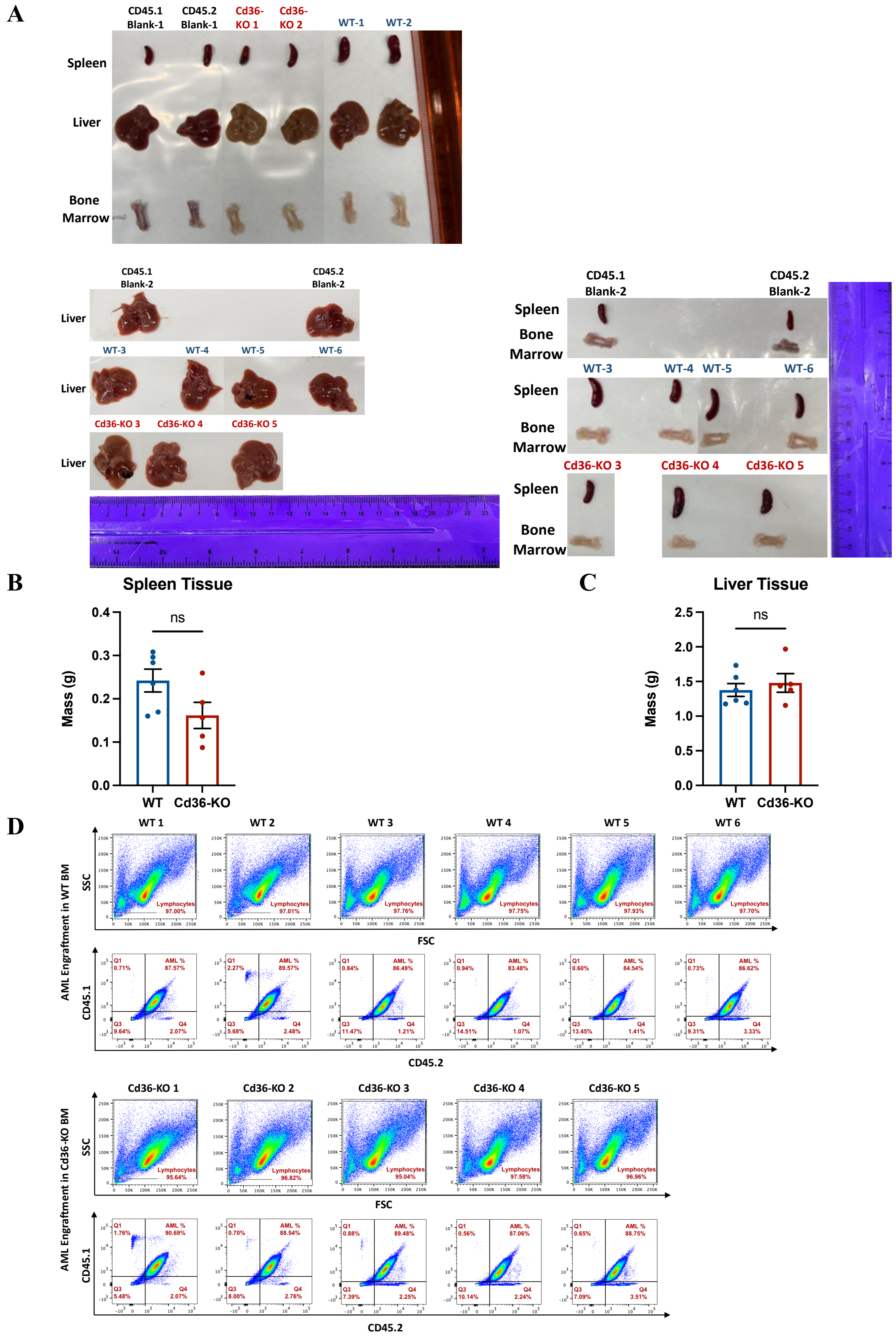

E

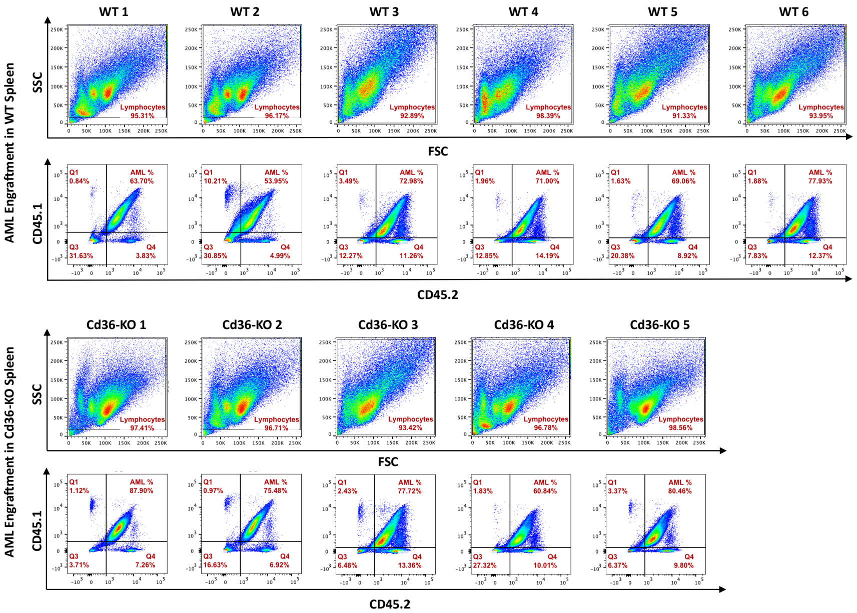
AML Engraftment in WT Spleen
WT 1
WT 2
WT 3
WT 4
WT 5
WT 6
SSC
FSC
Lymphocytes 95.31%
Lymphocytes 96.17%
Lymphocytes 92.89%
Lymphocytes 98.39%
Lymphocytes 91.33%
Lymphocytes 93.95%
CD45.1
CD45.2
Q1 0.84% AML % 63.70% Q3 31.63% Q4 3.83%
Q1 10.21% AML % 53.95% Q3 30.85% Q4 4.99%
Q1 3.49% AML % 72.98% Q3 12.27% Q4 11.26%
Q1 1.96% AML % 71.00% Q3 12.85% Q4 14.19%
Q1 1.63% AML % 69.06% Q3 20.38% Q4 8.92%
Q1 1.88% AML % 77.93% Q3 7.83% Q4 12.37%
AML Engraftment in Cd36-KO Spleen
Cd36-KO 1
Cd36-KO 2
Cd36-KO 3
Cd36-KO 4
Cd36-KO 5
Lymphocytes 97.41%
Lymphocytes 96.71%
Lymphocytes 93.42%
Lymphocytes 96.78%
Lymphocytes 98.56%
Q1 1.12% AML % 87.90% Q3 3.71% Q4 7.26%
Q1 0.97% AML % 75.48% Q3 16.63% Q4 6.92%
Q1 2.43% AML % 77.72% Q3 6.48% Q4 13.36%
Q1 1.83% AML % 60.84% Q3 27.32% Q4 10.01%
Q1 3.37% AML % 80.46% Q3 6.37% Q4 9.80%

F

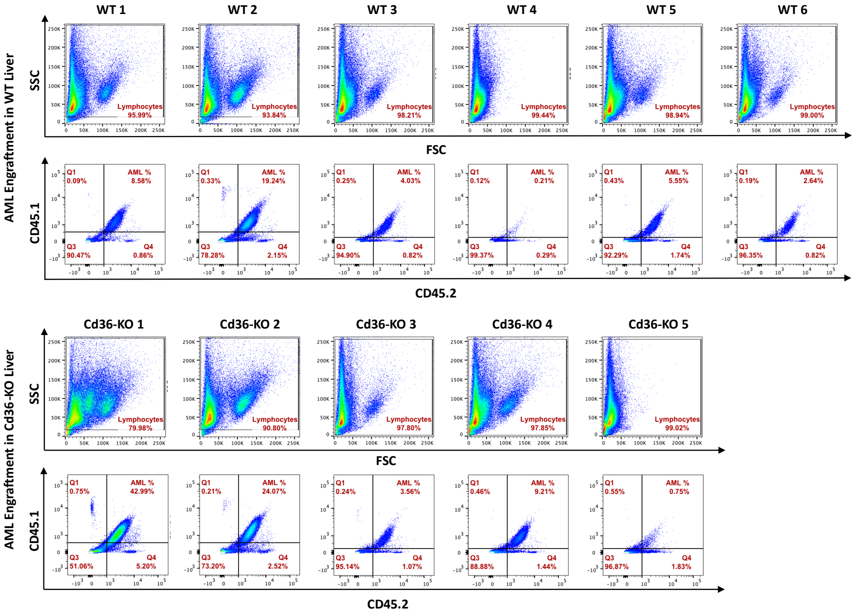
AML Engraftment in WT Liver
WT 1
WT 2
WT 3
WT 4
WT 5
WT 6
SSC
FSC
Lymphocytes 95.99%
Lymphocytes 93.84%
Lymphocytes 98.21%
Lymphocytes 99.44%
Lymphocytes 98.94%
Lymphocytes 99.00%
CD45.1
CD45.2
Q1 0.09% AML % 8.58% Q3 90.47% Q4 0.86%
Q1 0.33% AML % 19.24% Q3 78.28% Q4 2.15%
Q1 0.25% AML % 4.03% Q3 94.90% Q4 0.82%
Q1 0.12% AML % 0.21% Q3 99.37% Q4 0.29%
Q1 0.43% AML % 5.55% Q3 92.29% Q4 1.74%
Q1 0.19% AML % 2.64% Q3 96.35% Q4 0.82%
AML Engraftment in Cd36-KO Liver
Cd36-KO 1
Cd36-KO 2
Cd36-KO 3
Cd36-KO 4
Cd36-KO 5
Lymphocytes 79.98%
Lymphocytes 90.80%
Lymphocytes 97.80%
Lymphocytes 97.85%
Lymphocytes 99.02%
Q1 0.75% AML % 42.99% Q3 51.06% Q4 5.20%
Q1 0.21% AML % 24.07% Q3 73.20% Q4 2.52%
Q1 0.24% AML % 3.56% Q3 95.14% Q4 1.07%
Q1 0.46% AML % 9.21% Q3 88.88% Q4 1.44%
Q1 0.55% AML % 0.75% Q3 96.87% Q4 1.83%

G

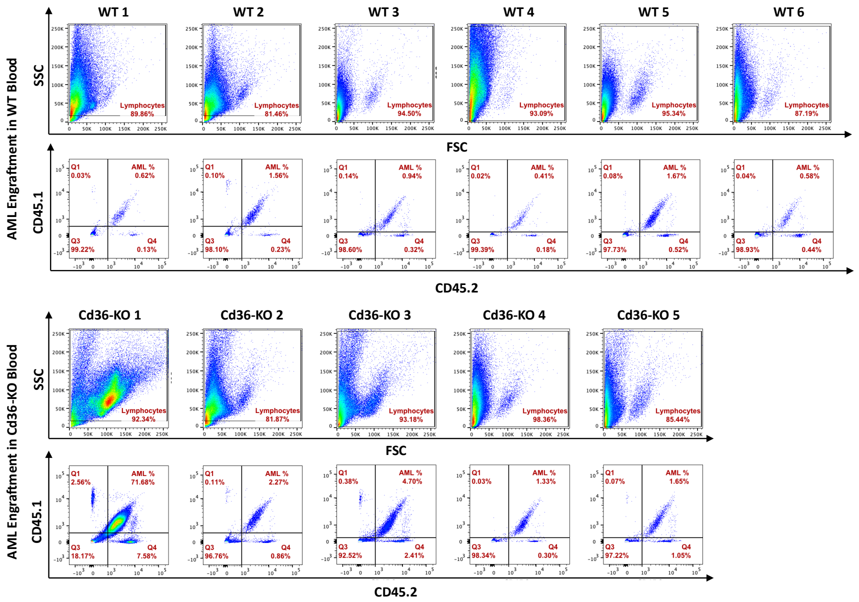

Figure S9. Cd36-KO mice exhibit similar AML engraftment with WT mice

(A) Spleens and livers were collected from Cd36-KO and WT mice engrafted with 5 x 10^6 FLT3-ITD/MLL-PTD mouse leukemic cells (n = 5 mice in KO; n = 6 mice in WT), one CD45.1 allele bearing blank mice, and one CD45.2 allele bearing blank mice.

(B-C) The weights of spleens and livers were measured, and data is presented as the mean of tissue mass from WT and KO group and each colored dot represents the mass of a single mouse tissue (n = 6 mice in WT group; n = 5 mice in Cd36-KO group). The differences between Cd36-KO and WT groups were analyzed by Mann-Whitney test (Abbreviation: ns, not significant).

(D-G) Representative flow cytometry result showing AML engraftment of FLT3-ITD/MLL-PTD mouse leukemic cells in the BM, spleen, liver, and blood cells of WT and Cd36-KO mice (n = 6 mice in WT group; n = 5 mice in Cd36-KO group).

Figure S10. Cd36-KO mice exhibit similar AML engraftment with WT mice

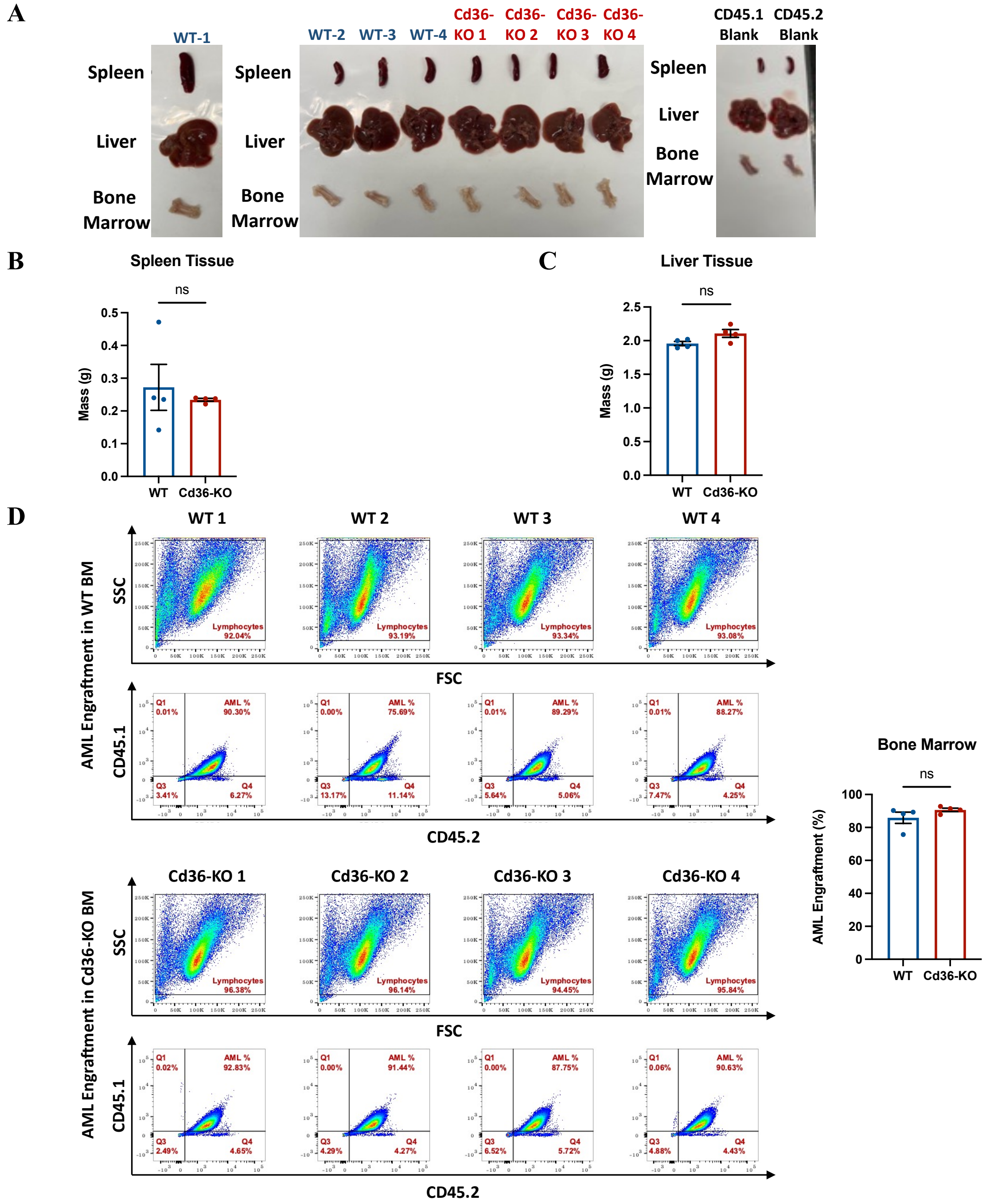

E

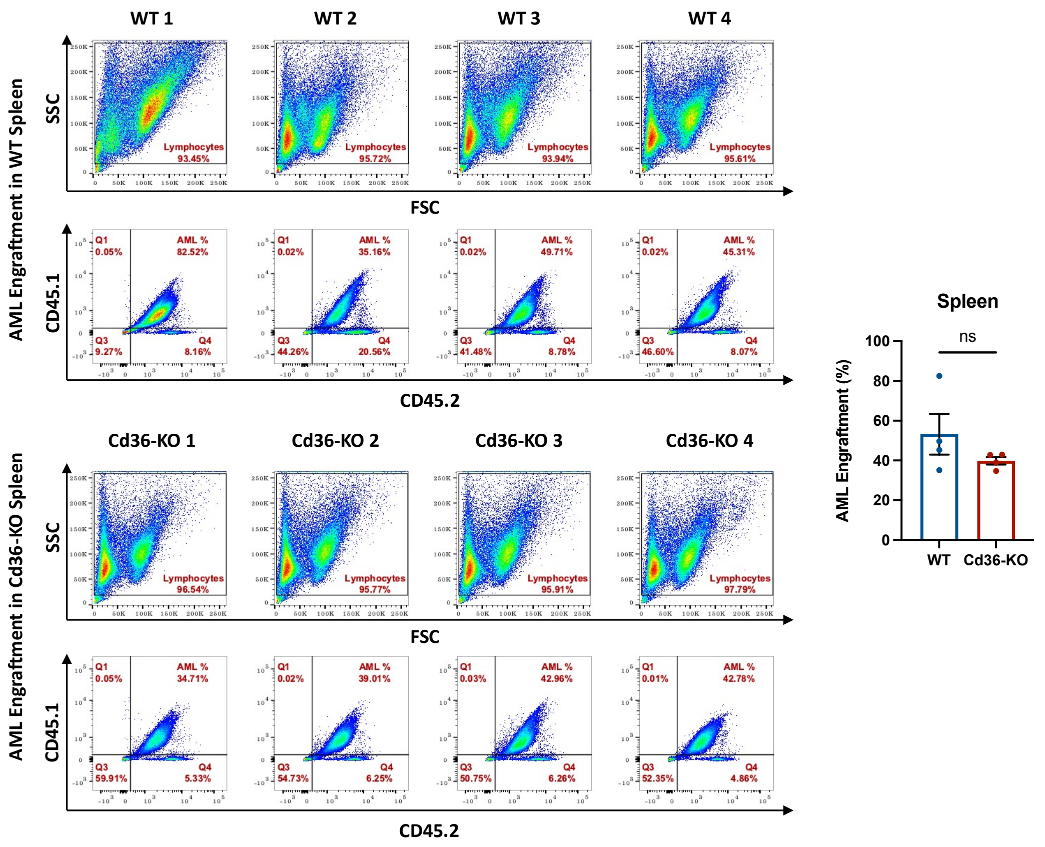

WT 1
WT 2
WT 3
WT 4
AML Engraftment in WT Spleen
SSC
FSC
Lymphocytes 93.45%
Lymphocytes 95.72%
Lymphocytes 93.94%
Lymphocytes 95.61%
CD45.1
CD45.2
Q1 0.05% AML % 82.52% Q3 9.27% Q4 8.16%
Q1 0.02% AML % 35.16% Q3 44.26% Q4 20.56%
Q1 0.02% AML % 49.71% Q3 41.48% Q4 8.78%
Q1 0.02% AML % 45.31% Q3 46.60% Q4 8.07%
Cd36-KO 1
Cd36-KO 2
Cd36-KO 3
Cd36-KO 4
AML Engraftment in Cd36-KO Spleen
Lymphocytes 96.54%
Lymphocytes 95.77%
Lymphocytes 95.91%
Lymphocytes 97.79%
Q1 0.05% AML % 34.71% Q3 59.91% Q4 5.33%
Q1 0.02% AML % 39.01% Q3 54.73% Q4 6.25%
Q1 0.03% AML % 42.96% Q3 50.75% Q4 6.26%
Q1 0.01% AML % 42.78% Q3 52.35% Q4 4.86%
Spleen
ns
AML Engraftment (%)
WT
Cd36-KO

F

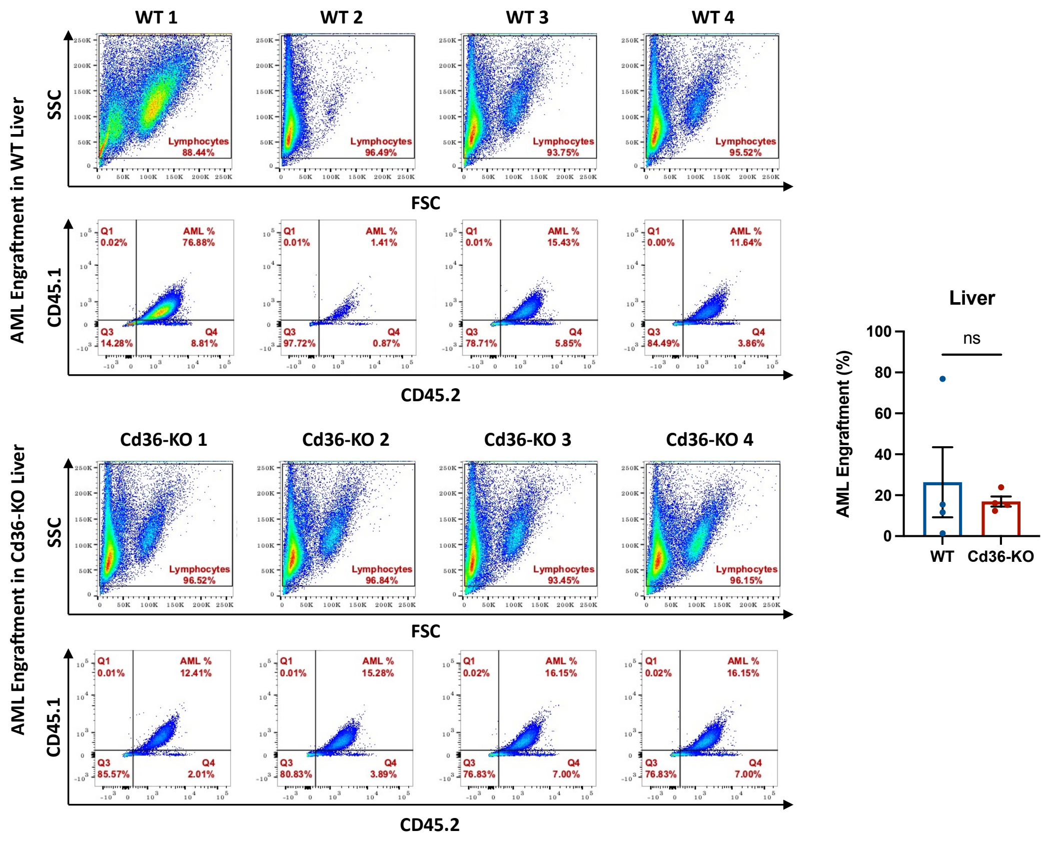

WT 1
WT 2
WT 3
WT 4
AML Engraftment in WT Liver
SSC
FSC
Lymphocytes 88.44%
Lymphocytes 96.49%
Lymphocytes 93.75%
Lymphocytes 95.52%
CD45.1
CD45.2
Q1 0.02% AML % 76.88% Q3 14.28% Q4 8.81%
Q1 0.01% AML % 1.41% Q3 97.72% Q4 0.87%
Q1 0.01% AML % 15.43% Q3 78.71% Q4 5.85%
Q1 0.00% AML % 11.64% Q3 84.49% Q4 3.86%
Cd36-KO 1
Cd36-KO 2
Cd36-KO 3
Cd36-KO 4
AML Engraftment in Cd36-KO Liver
Lymphocytes 96.52%
Lymphocytes 96.84%
Lymphocytes 93.45%
Lymphocytes 96.15%
Q1 0.01% AML % 12.41% Q3 85.57% Q4 2.01%
Q1 0.01% AML % 15.28% Q3 80.83% Q4 3.89%
Q1 0.02% AML % 16.15% Q3 76.83% Q4 7.00%
Q1 0.02% AML % 16.15% Q3 76.83% Q4 7.00%
Liver
ns
AML Engraftment (%)
WT
Cd36-KO

G

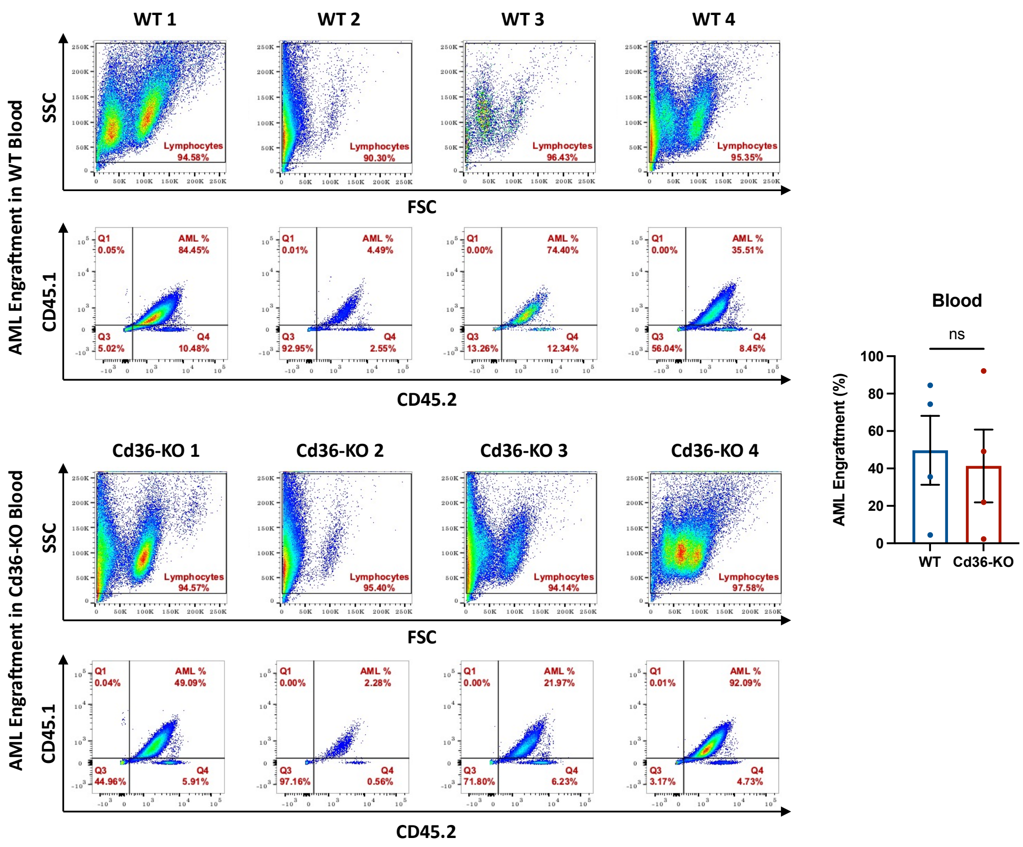

Figure S10. Cd36-KO mice exhibit similar AML engraftment with WT mice

(A) Spleens and livers were collected from Cd36-KO and WT mice engrafted with 1 x 10^6 FLT3-ITD/MLL-PTD mouse leukemic cells (n = 4 mice per group), one CD45.1 allele bearing blank mice, and one CD45.2 allele bearing blank mice.

(B-C) The weights of spleens and livers were measured, and data were presented as the mean of tissue mass from WT and KO group and each colored dot represents the mass of a single mouse tissue (n = 4 mice per group). The differences of tissue weight between Cd36-KO and WT groups were analyzed by Mann-Whitney test (Abbreviation: ns, not significant).

(D-G) Representative flow cytometry result showing AML engraftment and quantification of engraftment of FLT3-ITD/MLL-PTD mouse leukemic cells in the BM, spleen, liver, and blood cells of WT and Cd36-KO mice (n = 4 mice). The difference between groups were analyzed using unpaired t-test (Abbreviation: ns, not significant).
